# Meta-analysis reveals the vaginal microbiome is a better predictor of earlier than later preterm birth

**DOI:** 10.1186/s12915-023-01702-2

**Published:** 2023-09-25

**Authors:** Caizhi Huang, Craig Gin, Jennifer Fettweis, Betsy Foxman, Bizu Gelaye, David A. MacIntyre, Akila Subramaniam, William Fraser, Negar Tabatabaei, Benjamin Callahan

**Affiliations:** 1 Bioinformatics Research Center, North Carolina State University, Raleigh, 27606 USA; 2 Department of Population Health and Pathobiology, North Carolina State University, Raleigh, 27607 USA; 3 Department of Obstetrics and Gynecology, Virginia Commonwealth University, Richmond, 23284 USA; 4 Thomas Francis School of Public Health, University of Michigan, Raleigh, 27606 USA; 5 Department of Epidemiology, Harvard T. H. Chan School of Public Health, Boston, 02115 USA; 6 March of Dimes Prematurity Research Centre, Department of Metabolism, Digestion and Reproduction, Imperial College London, London, SW7 2AZ USA; 7 Obstetrics & Gynecology and Maternal-Fetal Medicine, University of Alabama at Birmingham, Birmingham, 35294 USA; 8 Departments of Obstetrics and Gynecology, University of Sherbrooke, Sherbrooke, J1K 2R1 USA; 9 Department of Pharmacology and Regenerative Medicine, University of Illinois College of Medicine, Chicago, 60612 USA

**Keywords:** Meta-analysis, Machine learning, Preterm birth, Vaginal microbiome

## Abstract

**Background:**

High-throughput sequencing measurements of the vaginal microbiome have yielded intriguing potential relationships between the vaginal microbiome and preterm birth (PTB; live birth prior to 37 weeks of gestation). However, results across studies have been inconsistent.

**Results:**

Here, we perform an integrated analysis of previously published datasets from 12 cohorts of pregnant women whose vaginal microbiomes were measured by 16S rRNA gene sequencing. Of 2039 women included in our analysis, 586 went on to deliver prematurely. Substantial variation between these datasets existed in their definition of preterm birth, characteristics of the study populations, and sequencing methodology. Nevertheless, a small group of taxa comprised a vast majority of the measured microbiome in all cohorts. We trained machine learning (ML) models to predict PTB from the composition of the vaginal microbiome, finding low to modest predictive accuracy (0.28–0.79). Predictive accuracy was typically lower when ML models trained in one dataset predicted PTB in another dataset. Earlier preterm birth (< 32 weeks, < 34 weeks) was more predictable from the vaginal microbiome than late preterm birth (34–37 weeks), both within and across datasets. Integrated differential abundance analysis revealed a highly significant negative association between *L. crispatus* and PTB that was consistent across almost all studies. The presence of the majority (18 out of 25) of genera was associated with a higher risk of PTB, with *L. iners*, *Prevotella*, and *Gardnerella* showing particularly consistent and significant associations. Some example discrepancies between studies could be attributed to specific methodological differences but not most study-to-study variations in the relationship between the vaginal microbiome and preterm birth.

**Conclusions:**

We believe future studies of the vaginal microbiome and PTB will benefit from a focus on earlier preterm births and improved reporting of specific patient metadata shown to influence the vaginal microbiome and/or birth outcomes.

**Supplementary Information:**

The online version contains supplementary material available at 10.1186/s12915-023-01702-2.

## Background

Preterm birth (PTB), defined as live birth prior to 37 complete weeks of gestation, is the primary cause of neonatal morbidity and mortality worldwide with an average PTB prevalence of around 11% [1, 2]. However, our current understanding of PTB is limited with no clear causative factor for the majority of PTBs [3]. One of the known risk factors of PTB is bacterial-related inflammation in gestational tissue [4, 5], including bacterial vaginosis (BV)―a polymicrobial alteration of the vaginal microbiome characterized by depletion of *Lactobacillus* species and overgrowth of typically strict anaerobes [6].

In the past decade, the development of high-throughput sequencing technologies has reformed the study of the human microbiome. High throughput sequencing of PCR-amplified marker genes (e.g., 16S rRNA gene) and shotgun metagenomic sequencing of total sample DNA are now widely used to measure the composition and functional potential of whole microbial communities. To date, at least 15 studies have used high-throughput sequencing to investigate the link between the vaginal microbiome and PTB, or preterm premature rupture of membranes (PPROM), which precedes between 30 and 40% of all PTB cases. All these studies employed a similar study design: (a) cohorts of pregnant women were recruited prospectively, (b) vaginal swabs were collected during the pregnancies, (c) birth outcomes (e.g., PTB) were recorded, (d) 16S rRNA gene sequencing was performed on a subset of women selected to meet pre-specified inclusion criteria and a target PTB to term birth (TB) ratio. However, these studies reported varied and sometimes inconsistent associations between the vaginal microbiome and PTB. For example, Romero et al. [7] found that vaginal microbial composition was not different in PTBs and TBs in a cohort of 90 pregnant women (88% African American; PTBs < 34 weeks). Digiulio et al. [8] reported that lower *Lactobacillus* and higher *Gardnerella* abundances in the vaginal microbiome were associated with a higher risk of PTB (55%+ White; PTBs < 37 weeks). Callahan et al. [9] replicated these findings in a study cohort drawn from the same population as Digiulio et al. [8], but not in a different cohort with a prior history of PTB (82% African American; PTBs < 37 weeks). Kindinger et al. [10] found a lack of *Lactobacillus crispatus* and *Lactobacillus iners* dominance were risk factors for PTB in a cohort of UK women (65% White; PTBs < 34 weeks). Fettweis et al. [11] reported lower *L. crispatus* abundance was a risk factor for PTB in a predominantly Black cohort of women (75% African American; PTBs < 37 weeks). These studies employed a variety of different sequencing methodologies, including targeting different regions of the 16S rRNA gene, and varied in how they included and reported spontaneous versus indicated preterm births.

The substantial heterogeneities between previous studies almost certainly contribute to the variation in reported associations between the vaginal microbiome and PTB. Relevant heterogeneities between studies exist across at least three axes: (1) study populations differed in important characteristics such as maternal race, BMI, and age; (2) the definition of PTB differed between studies, some of which considered only spontaneous PTBs or earlier PTBs (e.g., births at < 34 weeks of gestation) while others considered all PTBs; (3) different microbiome profiling methodologies were employed in each study including different DNA extraction methods and 16S rRNA gene primers. Based on previous work, it would be surprising if these heterogeneities did not introduce at least some variation in reported results. In terms of study population characteristics, Black women have a higher risk of PTB and BV compared to White women [12], and advanced maternal age is considered a risk factor for PTB [13]. In terms of study definitions of PTB, spontaneous PTB is usually related to preterm rupture of membranes (PPROM) or cervical dilation while indicated PTB is related to induced or cesarean section labor due to obstetrical complications [14]. Similarly, early PTB (< 32), moderate PTB ($\ge$ 32, < 34), and late PTB ($\ge$ 34, < 37) might be related to different causative factors, with an infectious etiology more commonly associated with early PTB [15]. In terms of study microbiome profiling methodologies, different DNA extraction methods can bias the detection of some microbial taxa over others [16], and it is known that standard primers for the V4 hypervariable region of the 16S rRNA gene have a higher sensitivity to the important *Gardnerella* genus compared with common V1-V3 primers [17, 18]. Finally, many of these studies have small sample sizes―12 cohorts included less than 50 women that experienced PTB, and 7 cohorts had an overall sample size of less than 100. Even in the absence of any other heterogeneities, a lack of power will result in inconsistencies in which results reach statistical significance across studies.

In a meta-analysis, the results from multiple studies regarding a common biological question are synthesized to achieve greater power and generalizability of the conclusions. For example, two meta-analyses of the gut microbiome and colorectal cancer (CRC) performed integrated analyses of multiple metagenomic CRC datasets to reveal consistent associations between the gut microbiome and CRC and to better understand the reproducibility of such associations across studies [19, 20]. Meta-analysis can help identify factors that cause inconsistencies between studies, aggregate signals across studies to improve power, and point the way towards improvements in future study design and analysis. Haque et al. [21] pooled 4 vaginal microbiome datasets to understand the temporal differences between the vaginal microbiome communities and found the diversity measures are significantly different between vaginal microbiomes sampled from women with term and preterm outcomes. However, they did not look into the role of specific genera or species. Kosti et al. [22] aggregated 5 longitudinal vaginal microbiome datasets with batch correction and reported several microbial genera as associated with PTB. However, they did not explore the heterogeneity cross-study and did not perform predictive analyses. Gudnadottir et al. [23] performed a network-based meta-analysis of 17 longitudinal vaginal microbiome datasets using community state types (CSTs). Their results supported the predictivity of preterm birth using the vaginal microbiome but did not build any prediction models and CSTs reduce the description of the vaginal microbiome to the identity of its most abundant member.

In this study, we performed a meta-analysis of 12 prospective case-control PTB datasets obtained by using 16S rRNA gene sequencing to measure the vaginal microbiome during pregnancy. All together, these 12 datasets included 2039 pregnant women, 586 of whom went on to deliver preterm. After re-processing the raw sequencing data using a consistent bioinformatics pipeline, we used a machine learning approach to investigate the predictability of PTB from the composition of the vaginal microbiome in each study [24, 25]. We evaluated cross-dataset reproducibility of PTB predictions from the vaginal microbiome and investigated PTB and study-specific factors that affected prediction accuracy. We explored the association between specific microbial taxa and PTB within and across studies. Finally, we synthesized these results into specific recommendations and cautions applicable to future study of the vaginal microbiome in PTB, and perhaps to the study of microbiomes in health and disease more broadly.

## Methods

### Data collection and availability

We used Google Scholar and PubMed to search the literature for studies published between 2014 and 2020 that used high-throughput 16S rRNA gene sequencing to characterize the vaginal microbiome during pregnancy in term and preterm births. We identified 15 such studies, all of which used some variation of a nested case-control study design drawn from larger cohorts of women who were prospectively enrolled and sampled during pregnancy. For inclusion in our meta-analysis, we required the raw sequencing to be available (either in a public archive or by request from the authors) and that the following metadata were also available: anonymized subject ID and gestational age at delivery. In total, this resulted in 12 datasets from independent cohorts of women that were included in this meta-analysis (Table 1). Datasets do not directly correspond to studies, since some studies contained multiple datasets from independent cohorts of women. Raw sequence data and metadata were downloaded from the NCBI Sequence Read Archive where possible, obtained from the supplementary materials of original papers, or requested from the authors. See Additional file 1: Table S1 for per-dataset details.

**Table 1:** Characteristics of the 16S rRNA gene sequencing and study cohorts for each dataset included in this meta-analysis

Dataset	Sequencing region	No. of subjects (preterm birth)	Sampling strategy	Maternal BMI**	Maternal age**	Maternal race***	Gestational age at sampling **	Reference
Brown2018 (Br)*	V1-V2	193 (157)	Longitudinal	25 (18–42)	33 (19–51)	43/50/84/0	22 (6–36)	[26–29]
Fettweis2019 (Fe)	V1-V3	135 (45)	Longitudinal	-	-	0/101/21/13	28 (3–41)	[11, 30]
Kindinger2017 (Ki)	V1-V3	161 (34)	Cross-sectional	25 (18–48)	33 (21–42)	27/30/104/0	16 (16–16)	[10, 31]
Romero2014 (Ro)	V1-V3	90 (18)	Longitudinal	30 (19–54)	25 (17–43)	1/79/5/5	26 (6–41)	[7, 32]
Stafford2017 (St)	V1-V3	133 (26)	Cross-sectional	-	29 (16–42)	4/8/110/11	24 (15–35)	[33, 34]
Digiulio2015 (Di)	V3-V5	40 (11)	Longitudinal	28 (18–50)	30 (19–41)	7/2/22/9	25 (1–40)	[8, 35]
Elovitz2019 (El)	V4	539 (107)	Longitudinal	-	-	0/399/115/25	− (16–28)	[36, 37]
Blostein2020 (Bl)	V4	125 (25)	Cross-sectional	26 (18–37)	28 (18–44)	-	9 ($<16$)	[38]****
ST_Callahan2017 (SC)	V4	39 (9)	Longitudinal	25 (18–51)	33 (25–42)	4/1/22/12	24 (2–41)	[9, 39, 40]
Subramaniam2016 (Su)	V4	38 (19)	Cross-sectional	25 (16–38)	22 (15–35)	0/18/20/0	− (21–25)	[41, 42]
Tabatabaei2019 (Ta)	V4	450 (94)	Cross-sectional	24 (13–51)	31 (20–44)	19/31/322/78	− (8–13)	[43]****
UAB_Callahan2017 (UC)	V4	96 (41)	Longitudinal	31 (16–73)	27 (17–38)	1/79/9/7	27 (11–41)	[9, 39, 40]

$^{*}$Dataset Brown2018 was combined from [26] and [27]

$$^{**}$$Maternal BMI, age, and gestational age at sampling were summarized as mean (range)

$$^{***}$$Maternal race was categorized as Asian/Black/White/other

$$^{****}$$Raw data from Blostein2020 and Tabatabaei2019 are available upon request

### Bioinformatics

DADA2 [44] was used to process the raw sequence data and infer the amplicon sequence variants (ASVs). The details of the DADA2 pipeline for each study can be found in Additional file 1: Table S2 and the Github repository associated with this manuscript (https://github.com/hczdavid/metaManuscript). To obtain comparable ASVs among datasets, we divided the datasets into two groups based on the region of the 16S gene that was sequenced (V1-V2 and V4) with five datasets in the V1-V2 group and seven datasets in the V4 group. Then, we truncated the original ASVs separately for each group to a common V1-V2 or V4 region in three steps: (1) align the original ASVs to the SILVA 132 reference database [45] using the mothur software [46], (2) identify the overlapping sequencing region common to all ASVs in the group using an alignment visualization tool (MSAviewer), and (3) truncate the original ASVs and remove alignment gaps using the extractalign and degapseq commands. Furthermore, we assigned the taxonomy levels to each truncated ASV using DADA2 with SILVA 132 reference database [45]. *Lactobacillus* species were assigned manually using BLAST against sequences from cultured *Lactobacillus* strains (Additional file 2: List S1).

### Data processing

Samples with total reads less than 100 were excluded from the analysis. The 25 core genera/species were obtained by the following steps: (1) For the V1-V2 and V4 groups of datasets, identify the per-group set of “common” ASVs (ASVs present in all datasets) and “top” ASVs (proportion larger than 0.1% in any dataset); (2) assign genus-level (species-level for *Lactobacillus*) taxonomy to all top or common ASVs from either group, yielding 34 unique genera/species; and (3) filter down to core genera/species using the following criteria: (i) average relative abundance > 0.1% in at least 5 datasets and (ii) prevalence > 10% in at least 5 datasets (Additional file 3: Fig. S1).

### Data transformation

Due to the arbitrary sequencing read depth of each sample, we first converted count data obtained from the DADA2 pipeline to proportional abundance data by dividing by the read depth of each sample. As the proportional abundance data does not account for the compositionality of the microbiome data, we further performed the centered log-ratio (CLR) transformation (with $10^{-6}$ pseudo-abundance) and compared the performance with proportional data in the ML framework and DA analysis. We found that data with CLR transformation have a similar prediction accuracy with the proportional abundance data using the ML model. The one-side Wilcoxon rank-sum test using CLR-transformed data or proportional data sometimes give an opposite direction of effect. For genera with low prevalence and low abundance, if CLR-transformed data is used to do analysis, their effects on preterm birth are mostly determined by the geometric mean of all genera, instead of their own abundance. However, if proportional data are used, their effects are only determined by their own abundance. In addition, we also included additive log-ratio (ALR), natural logarithm (log), and rank transformation methods in the ML framework comparison.

### Machine learning

A machine learning framework using random forest classifiers was employed for intra-dataset, cross-dataset, and leave-one-dataset-out (LODO) analysis. Intra-dataset analysis was performed on a single dataset using 5-fold cross-validation. Cross-dataset analysis was performed based on a pair of two datasets: one dataset as a training set and the other as a testing set. LODO analysis was performed by combining all datasets as a training set except one hold-out dataset as a testing set (see Fig. 2A). Given the predicted and true results, the area under the receiver operating characteristic curve (AUC) is calculated using the ‘pROC’ R package. The AUC from intra-analysis was averaged among 20 repetitions. We performed ten repetitions of intra-dataset, cross-dataset, and LODO analysis in the preterm birth subgroup analysis and calculated the average AUC. The random forest classifier was implemented using the ‘randomForest’ R package. We set the hyperparameter nTree = 1000 and tuned mtry using the ‘caret’ R package.

### Feature importance

We used the SHAP value from the random forest classifier to determine the feature importance. SHAP values that are large in absolute value indicate that the corresponding feature was influential in the machine learning prediction for the given sample. Here, positive SHAP values indicate that the feature value is associated with preterm birth whereas negative SHAP values indicate that the feature value is associated with term birth. We trained random forest models on each dataset using the scikit-learn implementation of a random forest classifier. For each dataset, we used 5-fold cross-validation with ten repetitions and calculated the SHAP values [47] of the validation data using the Tree SHAP algorithm [48] as implemented in the SHAP package. The features were ranked according to the importance of each study by comparing the mean absolute SHAP values.

### Statistical analysis

A one-sided Wilcoxon rank-sum test was used to perform the dataset-specific differential abundance analysis and implemented using the wilcox.test function in R. A generalized linear mixed model was used to do differential abundance analysis with a random effect for each study. Specifically, for each genus, we fitted the following model: $logit(P(Y=PTB)) =Genus+Race+BMI+Age + (1 | Study)$. $Genus=1$ if a genus is present based on an abundance threshold of 0.001 and 0 otherwise. *Race*, *BMI*, and *Age* represent the maternal race, BMI, and age, respectively. The generalized linear mixed model was implemented using the glmer function in the ‘lme4’ R package. Due to missing maternal race, BMI, and age in some datasets and non-significance of these covariates except self-reported Black race vs. White race ($p = 0.04$), we also fitted the model without adjusting these covariates.

We further performed a Bayesian analysis by assuming that (1) the log odds of the presence of a genus given preterm birth follow a uniform prior distribution for each dataset and (2) the odds ratio between PTB and TB has the same underlying true distribution for each dataset. We use a uniform prior distribution for the odds ratio for the first dataset and then calculate the posterior distribution. Then, we let the posterior distribution of the odds ratio from the first dataset be the prior distribution for the second dataset and update the posterior distribution. We repeated the process until the last dataset to obtain the final posterior distribution of the odds ratio. See Additional file 4: Supplementary Methods section for more details.

## Results

### Published studies of the vaginal microbiome in term and preterm births are highly heterogeneous

We searched the published literature for studies between 2014 and 2020 that used high-throughput 16S rRNA gene sequencing to characterize the vaginal microbiome during pregnancy in term and preterm births. We identified 15 such studies, all of which used some variation of a nested case-control study design drawn from larger cohorts of women who were prospectively enrolled and sampled during pregnancy. From these 15 studies, we identified 12 datasets from independent cohorts of women that were complete enough (raw sequencing data and sufficient metadata) for us to include in this meta-analysis (Table 1; Methods). In total, these datasets include 6891 vaginal microbiome samples from 2039 pregnant women, 586 of whom had preterm births. After excluding samples with total reads less than 100 (Methods), we have 2025 women (584 of them had PTB) in our datasets.

There was a large amount of heterogeneity in technical, clinical, and cohort characteristics among these studies of the vaginal microbiome in term and preterm births. The number of subjects in the datasets we included in our meta-analysis varied from a low of 38 to a high of 539, and the percentage of subjects who went on to have preterm births varied from a low of 20% to a high of 81%, respectively. In terms of gestational age at sampling, two datasets (Ta and Ki) contained samples only from the first trimester (0–13 weeks) or early second trimester (16 weeks). The Su and El datasets contained samples only from the second trimester (14–28 weeks). The other eight datasets contained samples across trimesters. Seven datasets are from longitudinal studies (Table 1), with a range from 1 to 41 samples per subject. The V1-V2 region of the 16S rRNA gene was sequenced in five of these datasets and the V4 gene region in the other seven. Four datasets had most participants self-report their race as Black and six datasets had a majority of participants self-report their race as White. Three datasets excluded late PTB ($\ge$ 34, < 37) or early TB ($\ge$ 37, < 39), while all other datasets included these two categories. Seven datasets only included spontaneous PTB and at least two other datasets included both spontaneous and indicated PTB. The distributions of gestational age at delivery and select population characteristics also varied substantially between datasets (Additional file 3: Figs. S2 and S3).

### A limited set of core taxa make up a vast majority of the vaginal microbiome

We used a consistent bioinformatic protocol based on the DADA2 tool and the Silva reference database to generate tables of taxonomically-assigned amplicon sequence variants (ASVs) from the raw 16S sequencing data for each dataset (Additional file 1: Table S1; Methods). To work at the highest level of resolution possible, datasets were partitioned into the V1-V2 or V4 groups based on which region of the 16S gene was sequenced. Within each group, ASVs were truncated to the “intersection” region contained within all sequenced amplicons, allowing an ASV table containing all datasets in the group to be constructed. For analyses using all datasets combined, taxonomic profiles at the genus level (with special species-level discrimination performed within the *Lactobacillus* genus) were merged into a single table.

**Fig. 1:**
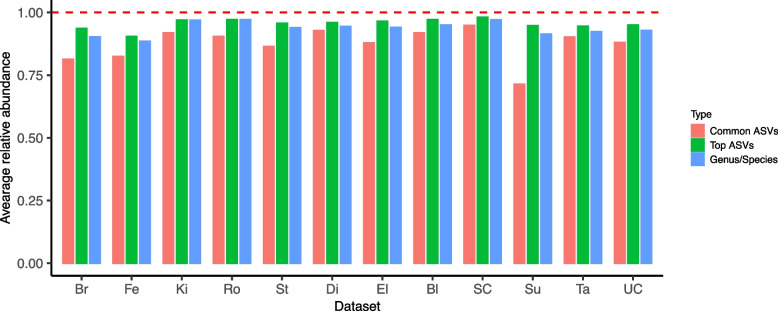
The average proportion of all sequencing reads in each dataset derived from the set of common ASVs (found in every dataset), top ASVs (proportion larger than 0.1% in any dataset), and core genus-level taxonomic features (abundance > 0.1% and prevalence > 10% for at least 5 datasets). Note that common and top ASV features were determined within the V1V2 and V4 dataset groups independently, as non-overlapping ASVs are not directly comparable (Methods)

A small number of taxa comprised a large majority of the vaginal microbiome in all studies included in our meta-analysis. In the V1-V2 group, we found 42 “common” ASVs that were present in all datasets and 157 “common” ASVs in the V4 group (Additional file 1: Table S3). These common ASVs constituted a large majority of the vaginal microbiome in every dataset in both the V1-V2 and V4 groups (71.3–94.8% of total reads). Another frequent strategy that is used to select a set of cross-dataset taxonomic features is to consider all taxa that appear above some abundance threshold in any dataset. Here, we defined “top” ASVs as those that had a relative abundance larger than 0.1% in any dataset. We found 172 top ASVs and 159 top ASVs in the V4 group and the V1-V2 group, respectively. This still modest number of ASVs constituted an overwhelming majority of the vaginal microbiome in every dataset (90.4– 98.0% total reads; 349–5479 ASVs). Finally, we also selected a set of “core” genera from the all-study table (both V1-V2 and V4 studies classified at the genus level, with *Lactobacillus* discriminated at the species level) using a hybrid filtering strategy that kept all genera present in at least 0.1% abundance and 10% prevalence in at least 5 datasets. Just 25 “core” genus-level taxa made up 88.4–97.1% of the total reads in every dataset (Fig. 1; Additional file 3: Figs. S1 and S4). Five genera (or *Lactobacillus* species) had particularly high average relative abundance (> 0.05) and prevalence (> 40%): *L. iners* (0.35, 87%), *L. crispatus* (0.29, 78%), *L. jensenii* (0.06, 57%), *L. gasseri* (0.057, 46%), and *Gardnerella* (0.054, 56%).

### The predictivity of preterm birth from the vaginal microbiome is low

**Fig. 2:**
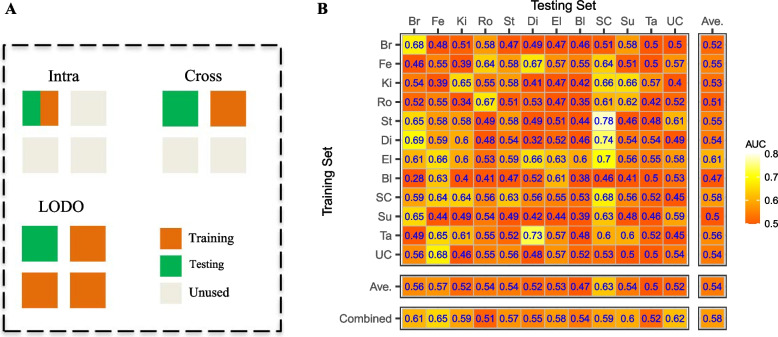
**A** A schematic of different analytical strategies using machine learning. Each square represents a different dataset, and squares are colored by how they are used to train or test the ML model. **B** The prediction accuracy, as measured by the AUC, for random forest ML models trained on the vaginal microbiome profiles (genus-level proportion data) in one dataset (rows) and tested in the same or a different dataset (columns). “Ave.” indicates the average AUC of each row (same training dataset) or each column (same testing dataset)

We employed a machine learning (ML) approach to assess the predictability of preterm birth outcomes from the genus-level composition of the vaginal microbiome. In order to assess the generalizability of ML predictions, we performed three types of ML analyses―intra-dataset analyses in which the ML model is trained and tested within a single dataset, cross-dataset analyses in which the ML model is trained on one dataset and tested on another, and leave-one-dataset-out (LODO) analyses in which 11 of 12 datasets are pooled together for training and testing is performed on the left-out dataset (Fig. 2A; Methods). Based on our evaluation of overall performance (Methods; Additional file 3: Figs. S5 and S6; Additional file 4: Supplementary Methods) and precedent in the microbiome field, we used the random forest classifier and either proportions or centered log-ratio (CLR) transformed abundances as our ML features. We used the area under the receiver operating characteristic curve (AUC) as our primary measure of ML prediction accuracy.

The predictivity of PTB from the vaginal microbiome varied substantially across studies. Intra-dataset AUCs ranged from 0.32 (no predictivity) to 0.68 (moderate predictivity) across the datasets we considered (Fig. 2B, values in the major diagonal). We did not discern an obvious pattern amongst the higher or lower-predictivity datasets. There was no clear increase in AUC with study size: low intra-study AUC (0.52) was obtained in the relatively well-powered Ta study (*n *= 450), while the highest intra-study AUC was in the relatively low power SC study (*n *= 39). The three studies (Br, Ro, and SC) with the highest AUCs (0.67–0.68) include cohorts with predominantly White and predominantly Black racial backgrounds, cohorts from California, Michigan, and the UK, and variously considered both all-cause and only spontaneous preterm births.

The predictivity of PTB by ML models trained on data from a different dataset was generally low. In the cross-dataset analyses―in which the ML model is trained on one dataset and then used to predict on a different dataset―only 22% of training/testing dataset pairs yielded AUCs larger than 0.6, and just 3% had AUCs larger than 0.7. There was some indication that certain datasets were easier, or harder, to predict PTB in than others (columns of Fig. 2B). AUCs for predictions in the SC dataset were greater than 0.6 for ML models trained on most other datasets, while the AUCs for predictions in the Ta study never exceeded 0.57 for any training dataset. The leave-one-dataset-out (LODO) analysis yielded slight increases in AUC in 10 out of 12 datasets compared to the average cross-dataset AUC. While similar in direction to the previous results in the meta-analysis of the gut microbiome in Thomas et al. [19] and Wirbel et al. [20], the magnitude of the increase in prediction accuracy obtained by pooling datasets together for ML training was much smaller than observed in those studies.

### Earlier preterm birth is more predictable than late preterm birth

Preterm birth is a syndrome with multiple causes, the relative importance of which may vary between different populations and between different sub-categories of preterm birth. One important subdivision of preterm birth is based on gestational age at delivery, with morbidity and mortality increasing sharply with earlier preterm births. Here, we used information available from each study about gestational age at delivery to define three PTB subgroups: (1) early preterm births (< 32 weeks), (2) early or moderate preterm births (< 34 weeks), and (3) late preterm births ($\ge$ 34 and < 37 weeks). Seven datasets had sufficient numbers of PTBs in each subgroup (8+) to include in our analysis. We employed intra-dataset and cross-dataset ML approaches to compare the predictivity of early, moderate, and late preterm births, with the control group set to full-term births ($\ge$ 39 weeks). To completely remove any potential effect of preterm birth sample size from the results, within each study we resampled the number of women in each PTB subgroup to the smallest number of women among all subgroups (Methods). Unfortunately, due to both a lack of available data in some studies and the exclusion of indicated preterm births in other studies, we were unable to perform a similar analysis comparing indicated and spontaneous preterm births.

**Fig. 3:**
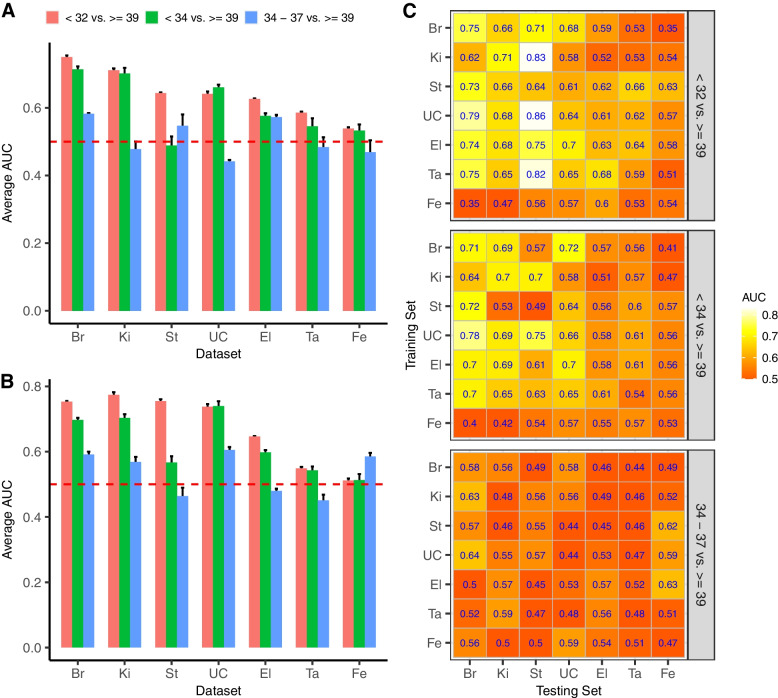
Assessment of prediction performance for different preterm birth groups using intra-dataset analysis (**A**), LODO analysis (**B**), and cross-dataset analysis (**C**) using CLR-transformed data. A resampling procedure is used to ensure each preterm birth group has the same sample size. The experiment is repeated 10 times and the average AUC and/or standard error are calculated. For each heatmap, diagonal AUC values are from intra-analysis, and off-diagonal values are from cross-analysis

Earlier preterm births were much easier to predict from the composition of the vaginal microbiome than were later preterm births. The accuracy for predicting late PTB was low to moderate in all intra-dataset and cross-dataset ML analyses (all AUC values $\le$ 0.65, Fig. 3C). In contrast, the accuracy for predicting early PTB was acceptable to good in most datasets (21 AUC values $\ge$ 0.65, 7 AUC values > 0.75, Fig. 3C). Classification accuracy for early-to-moderate PTB was intermediate, as expected. In most datasets, the intra-dataset and LODO accuracy for predicting early PTB were substantially better than late PTB (Fig. 3A and B), but the Fe dataset was an exception where classifier performance remained poor (AUC < 0.6) for all categories of PTB. Similar results were observed whether using proportions or CLR-transformed abundances as the features in the analysis (Additional file 3: Fig. S7).

### More resolved taxonomic features inconsistently affected the predictivity of PTB

We compared the prediction accuracy of our random forest ML models trained on the proportions of taxonomic features at different levels of resolution, from ASV up to Phylum (Table 2). In intra-dataset analysis, ASV level features had the overall best performance for the V1-V2 group, and there was an overall trend of decreasing AUC with increasingly broad taxonomic features. However, this trend was not evident in the V4 group (Table 2). In both the V1-V2 and V4 groups of datasets, the AUC measured in the cross-dataset and LODO analyses showed no clear trend with the breadth of the taxonomic features considered. Given the previous observation that earlier preterm birth is more predictable, we further investigated the prediction accuracy at different levels of feature resolution using only early PTB (< 32 weeks) and late-term birth ($\ge$ 39 weeks). In the V1-V2 group, we observed an overall trend of decreasing AUC with increasingly broad taxonomic features consistently in intra-dataset, cross-dataset, and LODO analyses. However, we did not see a similar trend in the V4 group of datasets (Additional file 1: Table S4).

**Table 2:** Average AUC values for different taxa level features

Analysis	V1-V2 group						V4 group					
	ASV	Genus	Family	Order	Class	Phylum	ASV	Genus	Family	Order	Class	Phylum
Intra	0.65	0.63	0.60	0.57	0.58	0.55	0.58	0.57	0.59	0.56	0.52	0.54
Cross	0.53	0.54	0.57	0.56	0.56	0.57	0.57	0.56	0.56	0.55	0.53	0.55
LODO	0.56	0.57	0.57	0.58	0.60	0.61	0.60	0.62	0.61	0.60	0.56	0.59

### The importance of microbial taxa to machine learning models varies across datasets

**Fig. 4:**
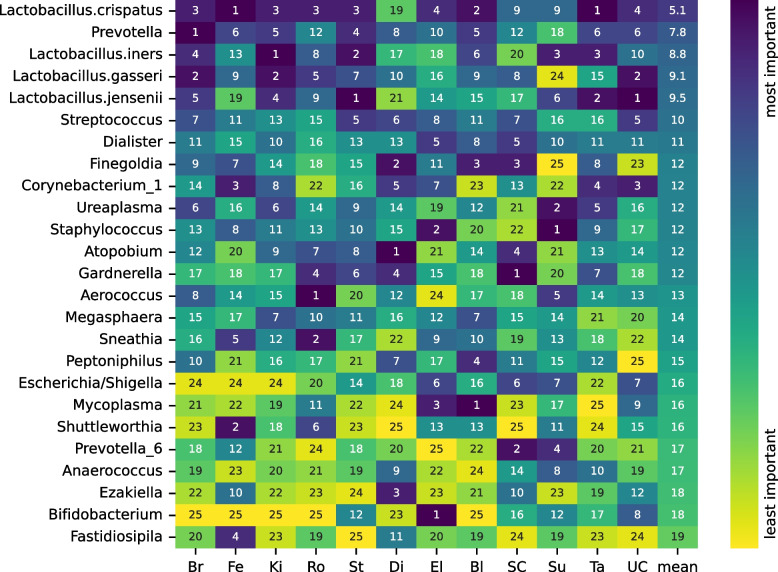
The feature importance ranking for genus-level taxonomic features (rows) in random forest ML models trained in different datasets (columns) using proportion data. Feature importance was quantified as the absolute SHAP value. Genera are ordered by their mean importance rank across all datasets

We further investigated the ML models by computing the importance of genus-level microbial features using SHAP (SHapley Additive exPlanations) values [47]. Figure 4 shows the feature ranking for each study for random forest models trained on proportional data. Averaged across all datasets, *L. crispatus* is the taxa that contributes most to the machine learning predictions, followed by *Prevotella* and *L. iners*. However, there is substantial variation in the importance of most taxa in ML models trained on different datasets. Consider, for example, *Finegoldia*. There are three datasets for which *Finegoldia* is among the three most important taxa and two studies for which it is among the three least important taxa. *Mycoplasma* is ranked as the most important taxa for the Bl dataset and the least important taxa for the Ta dataset. Further inconsistencies can be seen by examining the SHAP summary plot for each dataset (Additional file 3: Fig. S8). For most studies for which *Prevotella* is among the most important features, a high relative abundance of *Prevotella* is associated with preterm birth (see the Br, Ki, and St datasets). However, a high relative abundance of *Prevotella* is associated with term birth in the Bl dataset.

The varying importance of taxa in different datasets contributes to the lower prediction accuracy for PTB in the cross-dataset and leave-one-dataset-out (LODO) analyses. As an example, *Aerococcus* was the most important feature for the Ro dataset but was unimportant for all other datasets. Machine learning models trained on the Ro study heavily weight the abundance of *Aerococcus* in their predictions, even though *Aerococcus* is a poor predictor of preterm birth in other datasets. We trained ten random forests on the Ro dataset with and without including *Aerococcus* as a feature and made predictions on the other studies (Additional file 3: Fig. S9). For most studies, the AUC was higher when excluding *Aerococcus* from Ro, with notable improvements for the Fe, Di, SC, and Su studies.

### Emerging consensus associations between microbial genera and PTB

At an individual dataset level, differential abundance (DA) analysis using the one-sided Wilcoxon rank-sum test found associations between bacterial genera and PTB that largely agreed with the results reported in the original papers (Additional file 1: Table S5; Additional file 3: Fig. S10). This re-analysis confirms that for many genera, there is too much variation in the effect sizes and even direction of their association with PTB to draw robust conclusions from individual dataset analyses. This is unsurprising given the low power of many of these datasets. However, taxa with more consistent directions of effect did emerge when considering all the individual dataset DA results. In particular, *L. crispatus* was negatively associated with preterm birth in 10/12 datasets and *Gardnerella* was positively associated with preterm birth in 11/12 datasets.

**Fig. 5:**
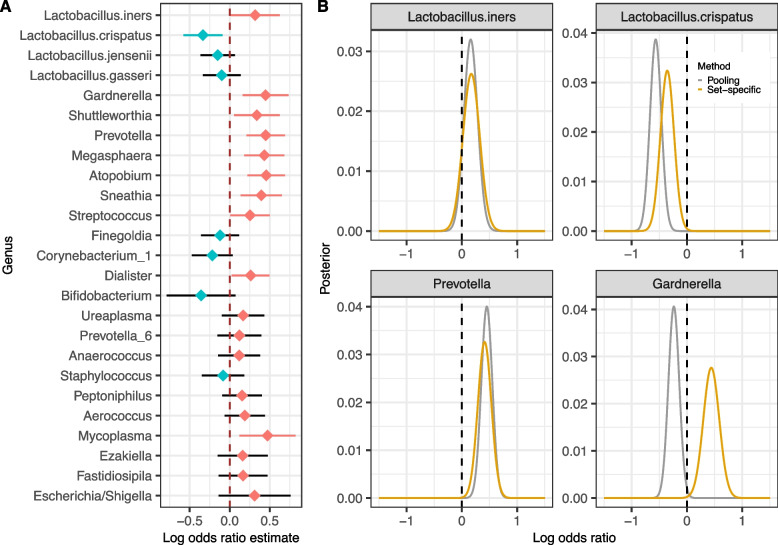
Cross-dataset differential abundance analysis. **A** Point estimates and 95% confidence intervals of the log odds ratio of a genus being present in preterm births relative to term births using a generalized linear mixed model. Presence was defined as a relative abundance greater than 0.001. The model included all 12 datasets and no population characteristic covariates. Point estimates less than 0 are shown as blue points and greater than 0 as red points. Confidence intervals less than 0 are shown as blue bars and greater than 0 as red bars. **B** Posterior distribution of log odds ratio using pooling and set-specific methods for four selected genera/species

In order to increase power, we performed an all-dataset differential prevalence analysis that also accounted for maternal age, BMI, and self-reported race. We created a prevalence (presence-absence) table at the genus level for each study by defining a genus as present in a sample if its proportion was greater than 0.1%. We fit a generalized linear mixed model (GLMM) to estimate the odds ratio between a genus is present versus absent with dataset-specific random effects separately for each genus (Fig. 5; Methods; Additional file 3: Fig. S11). The presence of three *Lactobacillus* species―*L. crispatus*, *L. jensenii*, and *L. gasseri*―were associated with reduced risk of PTB, while the presence of *L. iners* was associated with increased risk of PTB. Based on unadjusted *p*-values at a 0.05 level, we observed significant associations of *L. iners* ($p = 0.043$) and *L. crispatus* ($p=0.007$). The presence of most non-*Lactobacillus* genera (18 out of 21; Fig. 5A; Additional file 1: Table S6) was associated with a higher risk of PTB, consistent with a higher-diversity “bacterial-vaginosis-like” vaginal microbiome being associated with PTB. There were significant positive associations between PTB and the presence of *Gardnerella* ($p = 0.002$), *Shuttleworthia* ($p = 0.02$), *Prevotella* ($p = 0.0002$), *Megasphaera* ($p = 0.0007$), *Atopobium* ($p = 0.0001$), *Sneathia* ($p = 0.003$), *Streptococcus* ($p = 0.04$), *Dialister* ($p = 0.03$), and *Mycoplasma* ($p = 0.008$). We further investigated these associations in PTBs subdivided into early, moderate, and late subgroups as previously described (see the “Methods” section). We found that the associations between PTB and *L. iners*, *L. crispatus*, and *Prevotella* were stronger, i.e., had larger effect sizes and were more statistically significant, in earlier PTB than in late PTB (Additional file 3: Fig. S12).

### Ignoring dataset-specific characteristics in microbiome analyses can cause false signals

We developed two related Bayesian analyses of the association between genus prevalence and PTB, one in which datasets were pooled together as exchangeable equals (Pooling) and another in which the baseline rate at which a taxon was detected was allowed to vary among datasets (Set-specific). More specifically, we performed two Bayesian analyses in which the log odds of a genus to be present given preterm birth and the odds ratio of PTB relative to TB follow prior distributions. We calculated the posterior distribution of the odds ratio between PTB and TB using two methods: (1) Pooling, in which all datasets were combined as a large dataset, and (2) Set-specific, in which the posterior distribution of the association between genus prevalence and preterm birth was sequentially updated by application to each dataset while allowing for a dataset-specific baseline rate at which that genus was detected. Consistent with the GLMM results reported above, in the set-specific method, 18 genera had a maximum a posteriori (MAP; mode of the posterior distribution) value of their odds larger than 0 (i.e., a positive association between their presence and PTB) and seven genera had MAP smaller than 0 (Additional file 3: Fig. S13). Meaningful differences emerged when accounting for dataset-specific detection rates in some genera. For example, the set-specific method estimated a significant and positive association between the presence of *Gardnerella* and PTB, while the pooling method estimated a significant and negative association (Fig. 5B). Further inspection of this result revealed different detection rates of *Gardnerella* by most of the V1-V2 and the V4 studies. In Additional file 3: Fig. S1B, for example, we observed that the prevalence of *Gardnerella* at three V1-V2 studies (Br: 10%, Ki: 7%, St: 25%) is much lower than in all V4 studies. This result suggests that accounting for dataset-specific detection rates might be important when aggregating results across microbiome studies.

## Discussion

The identification of robust associations between host-associated microbiomes and health outcomes remains an elusive goal in many areas of microbiome research, as highlighted by many examples of specific associations that did not reproduce across studies [9, 20, 49]. There are several potential reasons for this. Most microbiome studies to date have been underpowered when considering the substantial temporal and inter-individual variability of host-associated microbial communities. Rapid progress in the laboratory and computational methods used to study microbiomes means that any two microbiome studies likely used measurement protocols different enough to make their results quantitatively incomparable [50–52]. The interaction between microbiomes and the host is mediated by poorly understood, and hence largely unrecorded, environmental and individual factors and myriad other challenges that are generic beyond microbiome studies, such as differences between study populations and the criteria used to define cases and controls. With these challenges in mind, we used a machine-learning and meta-analysis approach to study the relationship between the vaginal microbiome and preterm birth across 12 independent datasets consisting of taxonomic profiles obtained by 16S rRNA gene sequencing of vaginal swabs obtained during gestations that resulted in term and preterm births. Overall, this analysis revealed substantial heterogeneity in the relationship between vaginal microbiome measurements and preterm birth outcomes from different studies. Yet, generalizable results and lessons also emerged, perhaps most importantly the higher predictivity of the vaginal microbiome for earlier preterm births.

Earlier preterm births (< 32 weeks, < 34 weeks) were more predictable from the composition of the vaginal microbiome than were late preterm births (34–37 weeks). This pattern was observed across most of the seven datasets included in our analysis of PTB sub-categories. It was observed both in ML models that were trained and tested in the same dataset and in ML models that were trained in one dataset and tested in another. The two datasets (Ta and Fe) in which this pattern was not evident were also the two datasets in which PTB was the least predictable overall. A strength of this analysis is the strong control of between-study differences: comparisons are being made between early and late preterm births within a study, and the number of preterm births in each category per study is held constant. We believe these results support the prioritization of earlier preterm birth (< 34 weeks, or even earlier) in future studies of the relationship between the vaginal microbiome and PTB. Prioritization of earlier preterm births is consistent with their much higher morbidity and mortality. It is also supported by the arbitrariness of the 37-week cutoff, which can result in weak differences between term births (37–40 weeks) and the late preterm births (i.e., 34–37 weeks) that predominate in study cohorts targeting all preterm births.

Our meta-analysis of these 12 independent vaginal microbiome datasets increases the credibility of reported associations between *L. crispatus* and reduced risk of preterm birth, reported associations between *Gardnerella* and *Prevotella* and increased risk of preterm birth, and the different roles played by *L. iners* compared to other vaginal *Lactobacilli*. The most consistent finding across individual datasets was the negative association between the relative abundance of *L. crispatus* and preterm birth: 10 out of 12 datasets showed the same direction of effect. In 6 of these, the association had a raw *p*-value < 0.05. In our machine learning models, *L. crispatus* had the highest average importance across all studies for predicting PTB. When considering all datasets together, the association between the presence of *L. crispatus* and reduced risk of preterm birth was highly significant ($p = 0.007$) and was stronger for earlier preterm births. The association between *L. iners* and preterm birth was different from the other vaginal *Lactobacilli*: *L. iners* was associated with increased PTB risk in most individual studies, across all studies considered together, and more significantly so in earlier preterm births. Although the presence of several genera was associated with a higher risk of preterm birth when considering all studies together, *Gardnerella* was the genus most consistently associated with a higher risk of preterm birth at the individual-dataset level. *Prevotella* was the second most important taxa on average across our machine learning models. When all studies were considered together, it had the second most significant association with PTB and even stronger effect size and statistical significance in earlier preterm birth.

Two example taxa, *Aerococcus* and *Gardnerella*, demonstrate the important ways that differences in taxon-specific detection rates across studies can alter measured associations between the microbiome and preterm birth. In the cross-dataset meta-analysis, *Aerococcus* comprised a small to vanishing fraction of the vaginal microbiome and the presence of *Aerococcus* was marginally associated with higher PTB risk. In contrast, in the Ro dataset, *Aerococcus* was significantly associated with decreased PTB risk and was detected at a significantly higher baseline rate. ML models trained on Ro have *Aerococcus* as the most important genus for predicting PTB, whereas *Aerococcus* has little to no importance in models trained on other datasets. The importance of *Aerococcus* in Ro-trained models reduces their prediction accuracy in other datasets; the cross-dataset accuracy of models trained on Ro is higher when the *Aerococcus* feature is removed prior to training. We do not know what is driving this significant difference in the detection rate of *Aerococcus*, it could reflect real differences between the populations studied in Ro versus other studies, or it could reflect methodological differences such as a higher detection efficiency for *Aerococcus* of the primer mixture used in the Ro study. In another example, it has been known for some time that common V1 primers used in several studies here do not effectively amplify *Gardnerella* or the related *Bifidobacterium* [7, 17]. Consistent with this, we observed much lower proportions of *Gardnerella* in the V1-V2 studies, especially the Br, Ki, and St studies, that did not supplement their primer mixtures to detect *Gardnerella* better. Left unaddressed, this interfered with the cross-study estimation of the association between *Gardnerella* presence and preterm birth. Naively pooling the samples from all studies together led to the estimation of a negative association between *Gardnerella* and preterm birth. However, when our modeling incorporated study-specific differences in detection rates, *Gardnerella* was found to be significantly positively associated with preterm birth, in line with most reports in the literature.

The complex heterogeneities between different datasets significantly impede obtaining robustly generalizable results. The relative paucity of available subject metadata did not allow for post hoc control of many individual characteristics thought to modulate the vaginal microbiome, PTB risk, or both. Although maternal race, age, BMI, and gestational age at delivery were available from most datasets, several other important metadata were only available in some or a few datasets. For example, the definition of spontaneous vs. indicated PTB was only available for three datasets, while two datasets reported mixed spontaneous or indicated PTB. Prior history of preterm birth, which is a known risk factor for PTB, was only recorded for three datasets. Other complications such as pre-eclampsia and gestational diabetes, which are associated with a higher risk of PTB, were also underreported. Data on feminine hygiene practices such as douching was unavailable for several studies and has recently been reported to alter the relationship between the vaginal microbiome and preterm birth in White women [53]. It is therefore important that future studies capture and report comprehensive and detailed patient metadata that permit deeper analyses of potential confounding [54].

When considering all-cause and all-type PTB, we found that the predictivity of PTB from the composition of the vaginal microbiome was low to modest. This should not be surprising. PTB is a syndrome with multiple causes, and it is highly unlikely that PTBs arising from different causal mechanisms, e.g., indicated PTB due to placenta previa versus spontaneous preterm labor due to intrauterine infection [55], will associate with similar patterns in the vaginal microbiome. Indeed, it is likely that some etiologies of preterm birth will have no measurable relationship with the vaginal microbiome, and their inclusion in ML models would restrict the predictive accuracy. However, prediction of well-defined subtypes of PTB from the vaginal microbiome with the moderate to high accuracy needed for clinical relevance may be achievable.

The cross-dataset and leave-one-dataset-out machine learning results highlight the importance of careful interpretation when evaluating the generalizability of machine learning classifiers trained on microbiome data and indicate that single-study examples of high AUC should be met with caution. Figure 2B presents a classifier trained on the Br dataset that performs moderately well when tested on withheld test data from the same study (AUC = 0.68). However, it performs much worse when tested on data from other studies (average AUC = 0.52). This same behavior can be seen in previous microbiome meta-analyses that explored cross-dataset predictivity using machine learning [19, 20]. However, such analyses can also highlight differences in the underlying microbiota-host interactions of different patient cohorts. The Br dataset was enriched for PTB cases preceded by preterm prelabor rupture of the fetal membranes (PPROM), which is often associated with an infectious etiology [56]. Thus, despite producing a highly accurate within-study classifier, the performance of this classifier may not be maintained in other cohorts where population characteristics (e.g., PPROM prevalence) differ substantially. Methodological differences in DNA extraction or sequencing may also restrict cross-study classification accuracy. Consistent with this, we found that combining datasets for training ML models (the LODO analyses) did not meaningfully improve cross-dataset prediction accuracy versus using a single dataset for training. This is different than the meaningful improvements in accuracy obtained by pooling studies together reported for predicting colorectal cancer from the gut microbiome in Thomas et al. [19] and Wirbel et al. [20]. The reasons for this difference remain unclear but could arise from the higher heterogeneity of vaginal microbiome studies or even the PTB phenotype itself.

## Conclusions

Our work here has shown some of the challenges of integrating across microbiome studies, even those based on a common measurement technology (16S rRNA gene sequencing), but it has also shown the value of such work in identifying robust patterns that generalize beyond a single study. Based on our results, we make three suggestions for future studies of the vaginal microbiome and PTB: (1) earlier preterm birth should be prioritized, (2) the core genera discussed in this meta-analysis should be captured in future studies to reflect the community of the vaginal microbiome, and (3) comprehensive subject metadata should be recorded and made available to the wider research community, including specifically maternal race, age, BMI, prior history of PTB, the use of interventions designed to prevent preterm birth, gestational age at delivery, gestational age at the time of sample collection, and whether PTB was spontaneous or indicated. We are also hopeful that the application and integration of non-sequencing measurement technologies to the vaginal microbiome [57] will help bridge the gap between composition and function [58] and ultimately between observation and intervention.

### Supplementary information

**Additional file 1:** **Table S1.** Data availability and sequencing information for the datasets included in this meta-analysis. **Table S2.** The details of the DADA2 pipeline for the datasets included in this meta-analysis. **Table S3.** Number of features at ASV and other taxonomic levels. **Table S4.** Average AUC of using different feature levels using common feature table for early PTB ([MYLT] 32 weeks) and early or moderate PTB ([MYLT] 34 weeks) subgroups. **Table S5.** Summary of key reported genera/species in original studies. **Table S6.** Estimate of the odds ratio between genus is present relative to absent.

**Additional file 2:** **List S1.** Results of *Lactobacillus* species BLAST against sequences from cultured *Lactobacillus* strains.

**Additional file 3:** **Figure S1.** The relative abundance (A) and prevalence (B) of 34 genera/species either in common or top feature tables for both V1-V2 and V4 groups. The red number in the up-left shows the number of datasets that have relative abundance greater than 0.1% or prevalence greater than 10%. After filtering the genera using the following criteria (1) at least 5 datasets have the average relative abundance [MYGT] 0.1%; (2) at least 5 datasets have the average prevalence [MYGT] 10%, we excluded *Prevotella_7*, *Haemophilus*, *Lactobacillus.delbrueckii*, *Alloscardovia*, *Lactobacillus.amylovorus*, *Fusobacterium*, *Peptostreptococcus*, *DNF00809*, *Parvimonas* and kept another 25 genera/species for further analysis. **Figure S2.** The summary statistics of the population characteristics and gestational age at sampling. **Figure S3.** A: Distribution of gestational age delivery; B: Preterm birth type included in each dataset. **Figure S4.** The average prevalence and relative abundance of 25 core taxa across datasets. **Figure S5.** Assessment prediction performance of data transformation methods using the genus features. For intra-analysis, the area under the receiver operating characteristics curve (AUC) is obtained based on cross-validation within a dataset. For cross-analysis, the AUC value for a given dataset is the average of the AUCs using other datasets in V1-V2 or V4 group as the training set, and the dataset as the testing set. For LODO analysis, the AUC value for a given dataset is obtained using the combined dataset in V1-V2 or V4 group except the dataset as the training set and the study as the testing set. Rank row is calculated using the following steps (1) for each dataset, rank each method among all methods based on AUC values (larger AUC has a smaller rank); (2) calculate the average of the ranks across datasets for each method. **Figure S6.** Assessment prediction performance of classifiers using the genus features with CLR transformation. Rank row is calculated using the following steps (1) for each dataset, rank each method among all methods based on AUC values (larger AUC has a smaller rank); (2) calculate the average of the ranks across datasets for each method. **Figure S7.** Assessment of prediction performance for different preterm birth groups using intra-dataset analysis (A), combined-dataset analysis (B) and cross-dataset analysis (C) using proportional abundance data. **Figure S8.** SHAP summary plots for each study for random forests trained on genus-level proportional data. The five most important features for each study are shown. The data points are colored by the relative abundance of the taxa. Positive SHAP values indicate a higher likelihood of preterm birth and negative SHAP values indicate a lower likelihood of preterm birth in comparison to the average prediction. **Figure S9.** Average AUC with and without Aerococcus included as a feature for ten random forest models trained on genus-level CLR data from the Ro study and tested on all other studies. **Figure S10.** Dataset-specific differential abundance analysis for each genus/species using a one-sided Wilcoxon rank-sum test. A cell is in red color if the average relative abundance of a genus/species in term birth is larger than the average relative abundance in preterm birth, otherwise in blue color. The unadjusted *p*-value significant code: ‘***’: 0.001, ‘**’: 0.01, ‘*’: 0.05. **Figure S11.** Cross-dataset differential abundance analysis using a generalized linear mixed model with covariates. The genus and race effects were estimated using 11 datasets with a model including race covariate. The BMI and age effects were estimated using 8 datasets with a model including race, BMI and age. Point estimates less than 0 are shown as blue points and greater than 0 are shown as red points. Confident intervals less than 0 are shown as blue bars and greater than 0 are shown as red bars. **Figure S12.** Average estimates of log odd ratio for each PTB subgroup versus late term birth using the generalized linear mixed model. The asterisk shows the significant level of the combined *p*-values: *: *p*-value< 0.05; **: *p*-value< 0.01; ***: *p*-value< 0.001. **Figure S13.** Posterior distribution of log odds ratio using a Bayesian approach stepwise method. Maximum a posteriori (MAP) estimates less than 0 are shown as blue lines and larger than 0 are shown as red lines. The 95% credible intervals are shown in the left top corner.

**Additional file 4:** **Supplementary Methods.** Details about classifiers used in the meta-analysis and the Bayesian approach.

## Data Availability

The availability of the raw sequencing data included in the meta-analysis is summarized in the Additional file 1: Table S1. The processed data and analysis code can be found at https://github.com/hczdavid/metaManuscript.

## Abbreviations

PTB: Preterm birth
TB: Term birth
ASV: Amplicon sequence variant
AUC: Area under the receiver operating characteristic curves
ML: Machine learning
LODO: Leave-one-dataset-out
CLR: Centered log-ratio
SHAP: SHapley additive exPlanations
DA: Differential abundance
GLMM: Generalized linear mixed model
MAP: Maximum a posteriori
