# Supplementary material for: Meta-analysis reveals the vaginal microbiome is a better predictor of earlier than later preterm birth
Source: BMC Biol. 2023 Sep 25;21:199. doi: 10.1186/s12915-023-01702-2 (PMC10518966; doi:10.1186/s12915-023-01702-2)
Supplement: Supplementary file 1 — Additional file 1: Table S1. Data availability and sequencing information for the datasets included in this meta-analysis. Table S2. The details of the DADA2 pipeline for the datasets included in this meta-analysis. Table S3. Number of features at ASV and other taxonomic levels. Table S4. Average AUC of using different feature levels using common feature table for early PTB ([MYLT] 32 weeks) and early or moderate PTB ([MYLT] 34 weeks) subgroups. Table S5. Summary of key reported genera/species in original studies. Table S6. Estimate of the odds ratio between genus is present relative to absent. [file 12915_2023_1702_MOESM1_ESM.docx]

**Additional file 1: Supplementary Tables**

**Table S1**. Data availability and sequencing information for the datasets included in this meta-analysis

| **Dataset** | **Sequence data** | **Metadata** | **Sequencing**  **Platform (Region)** | **Primer** | **If paired** | **Which used?** |
| --- | --- | --- | --- | --- | --- | --- |
| Brown2018 (Br)* | ENA (PRJEB30642 and PRJEB21325) | From authors | Illumina Miseq (V1-V2) | 28F/388R | Yes | Forward |
| Fettweis2019 (Fe) | SRA (PRJNA326441) | dbGap (phs001523) | Illumina Miseq (V1-V3) | - | Yes | Forward |
| Kindinger2017 (Ki) | ENA (PRJEB11895 and PRJEB12577) | From authors | Illumina Miseq (V1-V3) | 28F/519F | Yes | Forward |
| Romero2014 (Ro) | SRA (PRJNA242473) | SRA (PRJNA242473) | 454 amplicon Seq (V1-V3) | 27F/534R | No | Forward |
| Stafford2017 (St) | SRA (SRP065627) | Suppl. material | 454 amplicon Seq (V1-V3) | 27F/519R | No | Forward |
| Digiulio2015 (Di) | SRA (SRP288562) | From authors | 454 amplicon Seq (V3-V5) | 338F/906R | No | Forward |
| Elovitz2019 (El) | dbGap (phs001739) | dbGap (phs001739 | Illumina Hiseq (V3-V4) | 319F/806R | Yes | Forward and reverse |
| Blostein2020 (Bl) | From authors | From authors | Illumina Treseq (V4) | 515F/806R | Yes | Forward and reverse |
| ST_Callahan2017 (SC) | Suppl. material | Suppl. material | Illumina Hiseq (V4) | 515F/806R | Yes | Forward and reverse |
| Subramaniam2018 (Su) | SRA (PRJNA600021) | SRA (PRJNA600021) | Illumina Miseq (V4) | 515F/806R | Yes | Forward |
| Tabatabaei2019 (Ta)** | From authors | From authors | Illumina (V4) | 515F/806R | - | - |
| UAB_Callahan2017 (UC) | Suppl. material | Suppl. material | Illumina Hiseq (V4) | 515F/806R | Yes | Forward and reverse |

** Dataset Brown2018 is combined of Brown, et al (2018) and Brown, et al (2019).

** For Tabatabaei2019, only the processed FASTA file is available to us. We re-created the FASTQ file using an arbitrary quality score.

**Table S2**. The details of the DADA2 pipeline for the datasets included in this meta-analysis.

| **Dataset** | **Pre-step to remove primer** | **Truncate length (truncLen)** | **trimLeft** | **maxEE** | **truncQ** |
| --- | --- | --- | --- | --- | --- |
| Brown2018 (Br) | No* | 245 | 19 | 2 | 2 |
| Fettweis2019 (Fe) | No | 260 | 20 | 2 | 2 |
| Kindinger2017 (Ki) | Yes | 250 | 0 | 2 | 2 |
| Romero2014 (Ro) | Yes | 450 | 0 | 2 | 2 |
| Stafford2017 (St) | Yes | 350 | 0 | 2 | 2 |
| Digiulio2015 (Di) | Yes | 400 | 0 | 2 | 2 |
| Elovitz2019 (El) | Yes | 270 | 0 | 2 | 2 |
| Blostein2020 (Bl) | No | 0 | 19/20 | 2 | 2 |
| ST_Callahan2017 (SC) | No | 235 | 10 | 2 | 2 |
| Subramaniam2018 (Su) | Yes | 250 | 0 | 2 | 2 |
| Tabatabaei2019 (Ta)** | No | 252 | 0 | 2 | 2 |
| UAB_Callahan2017 (UC) | No | 235 | 10 | 2 | 2 |

* Only some samples have primers included in the sequence data. See the DADA2 code at GitHub for details.

** For Tabatabaei2019, only the processed FASTA file is available to us. We re-created the FASTQ file using an arbitrary quality score.

**Table S3**. Number of features at ASV and other taxonomic levels.

| Feature  Level | V1-V2 group | | V4 group | |
| --- | --- | --- | --- | --- |
|  | Common | Top | Common | Top |
| Phylum | 6 | 8 | 9 | 10 |
| Class | 9 | 12 | 15 | 14 |
| Order | 10 | 20 | 24 | 23 |
| Family | 13 | 26 | 42 | 35 |
| Genus | 22 | 45 | 84 | 63 |
| ASV | 42 | 172 | 157 | 159 |

**Table S4**. Average AUC of using different feature levels using common feature table for early PTB (<32 weeks) and early or moderate PTB (<34 weeks) subgroups.

| PTB groups | Analysis | V1-V2 group (4 datasets) | | | | | | V4 group (3 datasets) | | | | | | |
| --- | --- | --- | --- | --- | --- | --- | --- | --- | --- | --- | --- | --- | --- | --- |
|  |  | ASV | Genus | Family | Order | Class | Phylum | ASV | Genus | Family | Order | Class | Phylum |  |
| < 32 weeks | Intra | 0.71 | 0.70 | 0.67 | 0.68 | 0.68 | 0.66 | 0.62 | 0.62 | 0.66 | 0.64 | 0.60 | 0.60 |  |
|  | Cross | 0.68 | 0.66 | 0.66 | 0.66 | 0.66 | 0.65 | 0.56 | 0.60 | 0.59 | 0.60 | 0.59 | 0.62 |  |
|  | LODO | 0.72 | 0.71 | 0.63 | 0.61 | 0.61 | 0.64 | 0.57 | 0.61 | 0.62 | 0.62 | 0.58 | 0.61 |  |
| < 34 weeks | Intra | 0.70 | 0.68 | 0.63 | 0.62 | 0.62 | 0.62 | 0.66 | 0.65 | 0.66 | 0.64 | 0.63 | 0.62 |  |
|  | Cross | 0.63 | 0.60 | 0.61 | 0.62 | 0.62 | 0.60 | 0.60 | 0.60 | 0.58 | 0.60 | 0.60 | 0.60 |  |
|  | LODO | 0.68 | 0.64 | 0.61 | 0.59 | 0.59 | 0.62 | 0.59 | 0.62 | 0.60 | 0.61 | 0.59 | 0.62 |  |

**Table S5**. Summary of key reported genera/species in original studies.

| Datasets | Key genera/species finding | |
| --- | --- | --- |
|  | Increasing abundance in PTB | Decreasing abundance in PTB |
| Br | Prevotella, Peptoniphilus, Streptococcus, Dialister | Lactobacillus |
| Fe | BVAB1, Sneathia amnii, TM7-H1, Prevotella | Lactobacillus crispatus |
| Ki | Lactobacillus iners | Lactobacillus crispatus |
| Ro | Not report | Not report |
| St | Lactobacillus jensenii | Lactobacillus crispatus/gasseri |
| Di | Gardnerella, Ureaplasma | Lactobacillus |
| El | Mobiluncus curtsii/mulieris and Sneathia sanguinegens | Not report |
| Bl | Not report | Not report |
| SC | Gardnerella | Lactobacillus crispatus |
| Su | Not report | Not report |
| Ta | Not report | Lactobacillus, Bifidobacterium |
| UC | Not report | Not report |

**Table S6**. Estimate of the odds ratio between genus is present relative to absent.

| Genus/Species | Estimate | 95% CI | p-value | adjusted p-value |
| --- | --- | --- | --- | --- |
| Lactobacillus.iners | 1.37 | [1.01, 1.87] | 0.0425 | 0.101 |
| Lactobacillus.crispatus | 0.72 | [0.56, 0.91] | 0.0070 | 0.029 |
| Lactobacillus.jensenii | 0.86 | [0.70, 1.07] | 0.1723 | 0.273 |
| Lactobacillus.gasseri | 0.91 | [0.72, 1.15] | 0.4080 | 0.425 |
| Gardnerella | 1.56 | [1.18, 2.08] | 0.0021 | 0.013 |
| Shuttleworthia | 1.40 | [1.06, 1.87] | 0.0199 | 0.062 |
| Prevotella | 1.57 | [1.23, 1.99] | 0.0002 | 0.003 |
| Megasphaera | 1.54 | [1.20, 1.98] | 0.0007 | 0.006 |
| Atopobium | 1.58 | [1.25, 1.99] | 0.0001 | 0.003 |
| Sneathia | 1.48 | [1.15, 1.92] | 0.0027 | 0.014 |
| Streptococcus | 1.29 | [1.01, 1.65] | 0.0443 | 0.101 |
| Finegoldia | 0.89 | [0.70, 1.12] | 0.3208 | 0.382 |
| Corynebacterium_1 | 0.81 | [0.62, 1.04] | 0.0947 | 0.197 |
| Dialister | 1.30 | [1.03, 1.65] | 0.0302 | 0.084 |
| Bifidobacterium | 0.70 | [0.45, 1.08] | 0.1041 | 0.200 |
| Ureaplasma | 1.18 | [0.91, 1.54] | 0.2128 | 0.305 |
| Prevotella_6 | 1.13 | [0.86, 1.48] | 0.3863 | 0.420 |
| Anaerococcus | 1.13 | [0.87, 1.46] | 0.3764 | 0.420 |
| Staphylococcus | 0.92 | [0.71, 1.20] | 0.5406 | 0.541 |
| Peptoniphilus | 1.17 | [0.91, 1.50] | 0.2196 | 0.305 |
| Aerococcus | 1.21 | [0.94, 1.55] | 0.1402 | 0.250 |
| Mycoplasma | 1.60 | [1.13, 2.28] | 0.0081 | 0.029 |
| Ezakiella | 1.18 | [0.86, 1.61] | 0.3094 | 0.382 |
| Fastidiosipila | 1.18 | [0.87, 1.60] | 0.2796 | 0.368 |
| Escherichia/Shigella | 1.36 | [0.87, 2.14] | 0.1744 | 0.273 |
