# Supplementary material for: Meta-analysis reveals the vaginal microbiome is a better predictor of earlier than later preterm birth
Source: BMC Biol. 2023 Sep 25;21:199. doi: 10.1186/s12915-023-01702-2 (PMC10518966; doi:10.1186/s12915-023-01702-2)
Supplement: Supplementary file 3 — Additional file 3: Figure S1. The relative abundance (A) and prevalence (B) of 34 genera/species either in common or top feature tables for both V1-V2 and V4 groups. The red number in the up-left shows the number of datasets that have relative abundance greater than 0.1% or prevalence greater than 10%. After filtering the genera using the following criteria (1) at least 5 datasets have the average relative abundance [MYGT] 0.1%; (2) at least 5 datasets have the average prevalence [MYGT] 10%, we excluded Prevotella_7, Haemophilus, Lactobacillus.delbrueckii, Alloscardovia, Lactobacillus.amylovorus, Fusobacterium, Peptostreptococcus, DNF00809, Parvimonas and kept another 25 genera/species for further analysis. Figure S2. The summary statistics of the population characteristics and gestational age at sampling. Figure S3. A: Distribution of gestational age delivery; B: Preterm birth type included in each dataset. Figure S4. The average prevalence and relative abundance of 25 core taxa across datasets. Figure S5. Assessment prediction performance of data transformation methods using the genus features. For intra-analysis, the area under the receiver operating characteristics curve (AUC) is obtained based on cross-validation within a dataset. For cross-analysis, the AUC value for a given dataset is the average of the AUCs using other datasets in V1-V2 or V4 group as the training set, and the dataset as the testing set. For LODO analysis, the AUC value for a given dataset is obtained using the combined dataset in V1-V2 or V4 group except the dataset as the training set and the study as the testing set. Rank row is calculated using the following steps (1) for each dataset, rank each method among all methods based on AUC values (larger AUC has a smaller rank); (2) calculate the average of the ranks across datasets for each method. Figure S6. Assessment prediction performance of classifiers using the genus features with CLR transformation. Rank row is calculated using the foll [file 12915_2023_1702_MOESM3_ESM.docx]

**Additional file 3: Supplementary Figures**


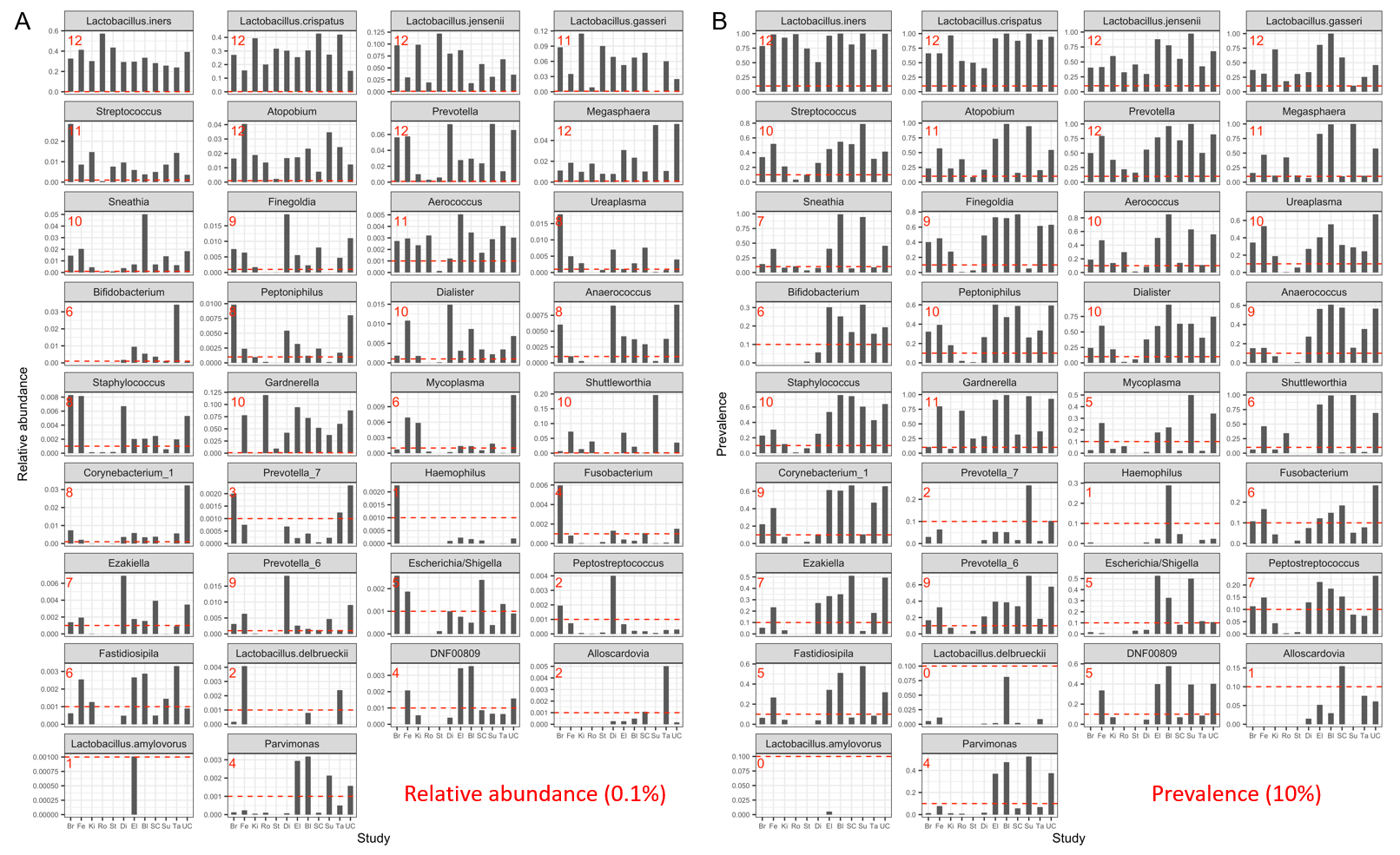


**Figure S1.** The relative abundance (A) and prevalence (B) of 34 genera/species either in common or top feature tables for both V1-V2 and V4 groups. The red number in the up-left shows the number of datasets that have relative abundance greater than 0.1% or prevalence greater than 10%. After filtering the genera using the following criteria (1) at least 5 datasets have the average relative abundance > 0.1%; (2) at least 5 datasets have the average prevalence > 10%, we excluded *Prevotella_7, Haemophilus, Lactobacillus.delbrueckii, Alloscardovia, Lactobacillus.amylovorus, Fusobacterium, Peptostreptococcus, DNF00809, Parvimonas* and kept another 25 genera/species for further analysis.


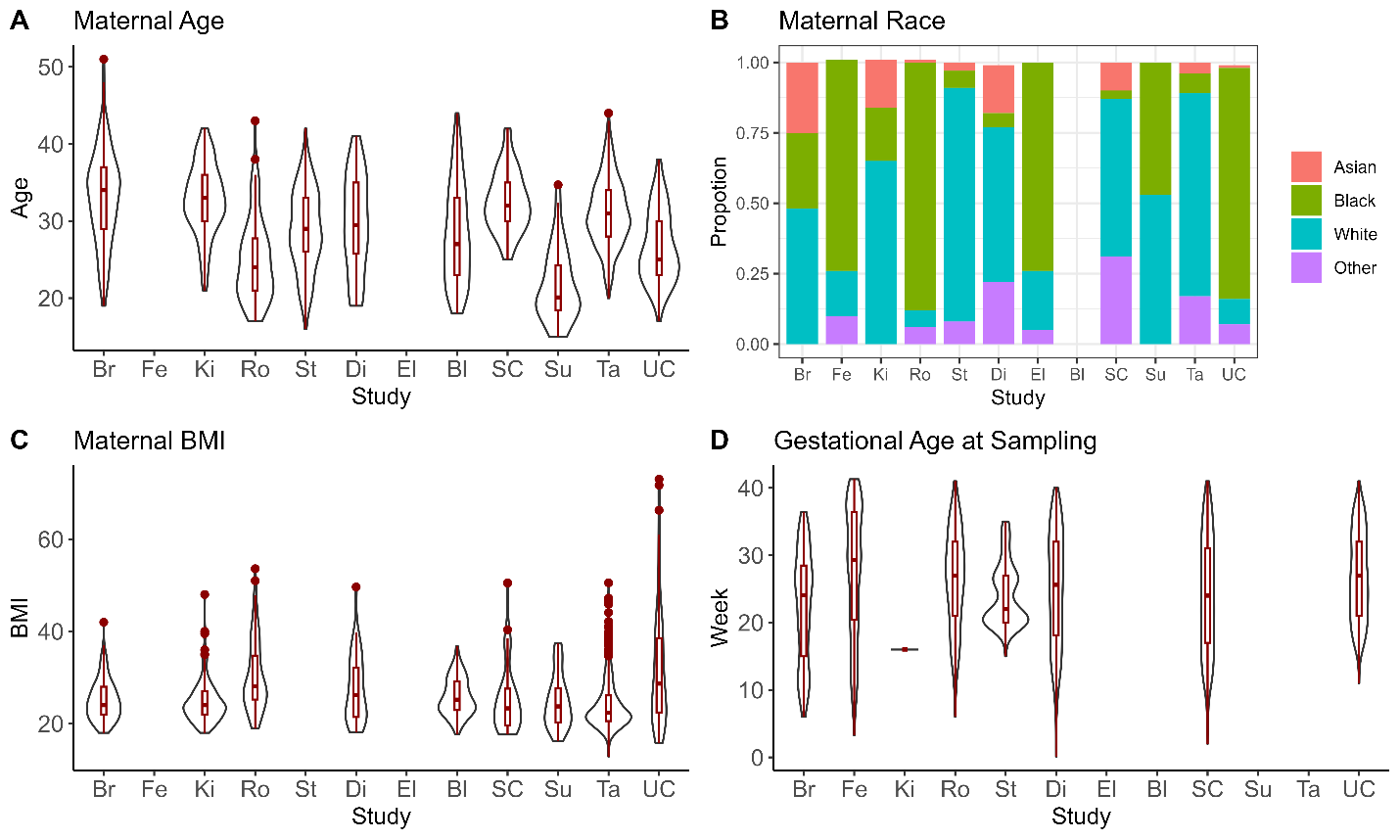


**Figure S2.** The summary statistics of the population characteristics and gestational age at sampling.


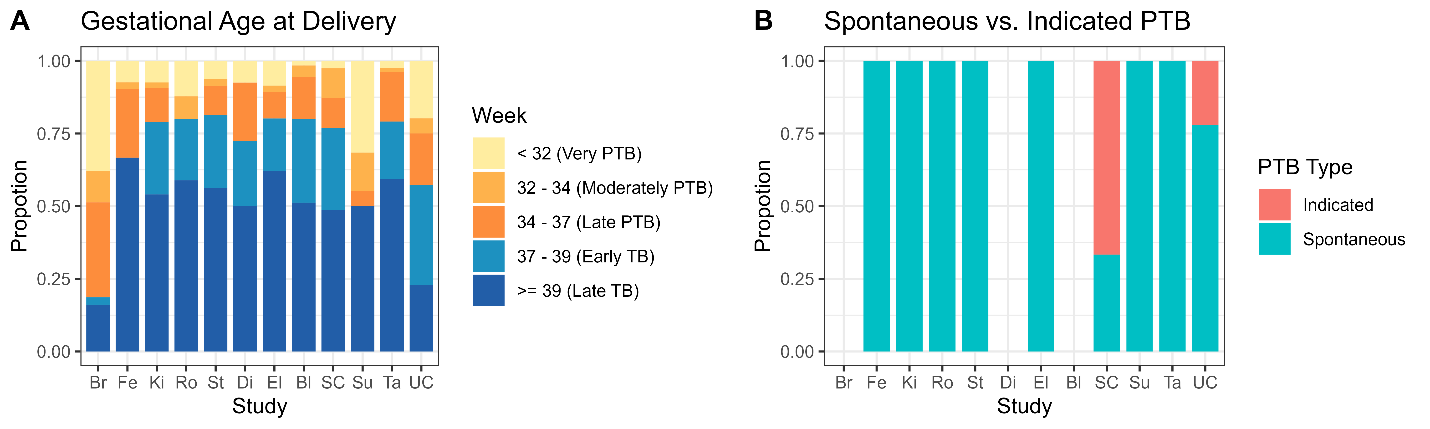


**Figure S3.** A: Distribution of gestational age delivery; B: Preterm birth type included in each dataset.


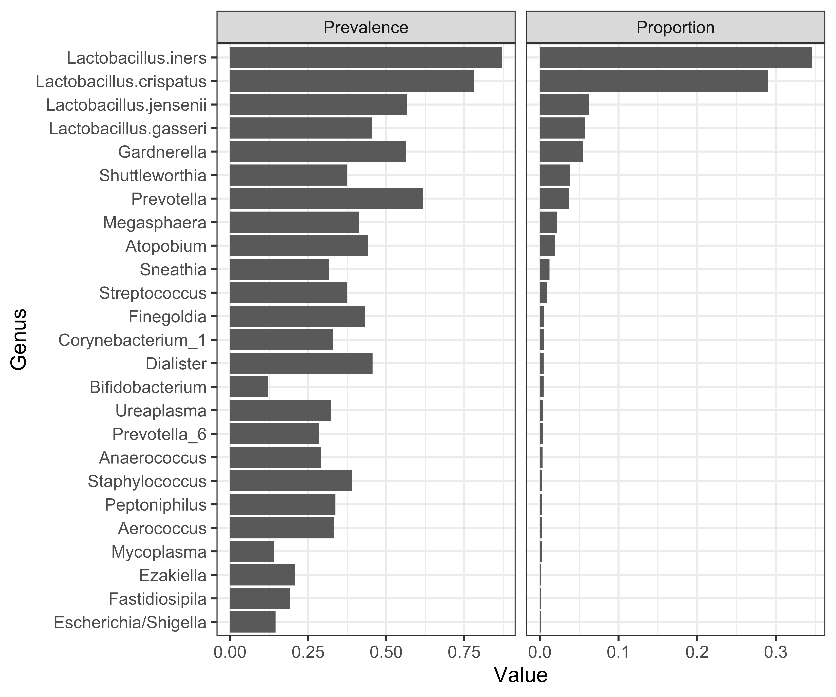


**Figure S4.** The average prevalence and relative abundance of 25 core taxa across datasets.


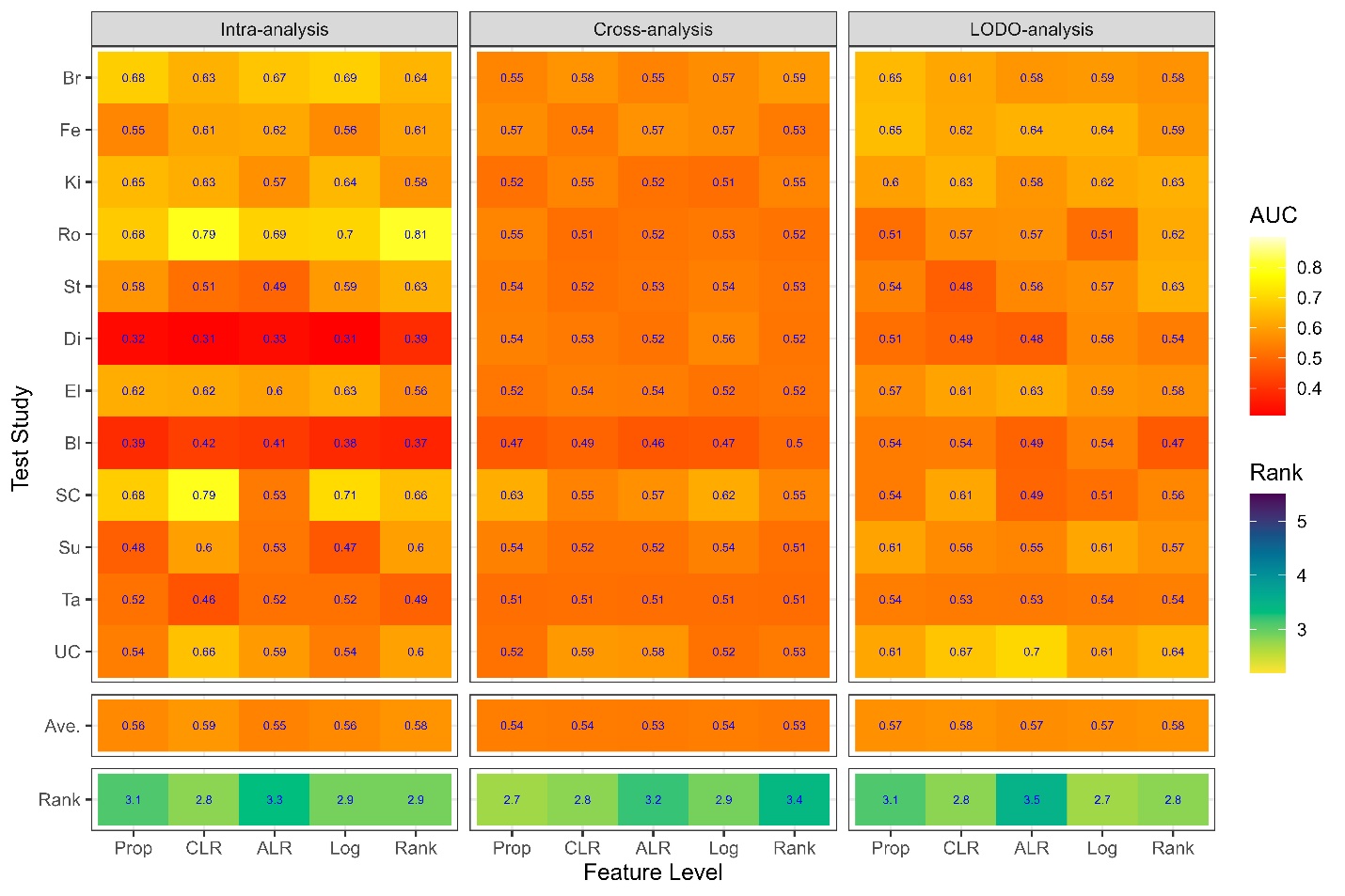


**Figure S5.** Assessment prediction performance of data transformation methods using the genus features. For intra-analysis, the area under the receiver operating characteristics curve (AUC) is obtained based on cross-validation within a dataset. For cross-analysis, the AUC value for a given dataset is the average of the AUCs using other datasets in V1-V2 or V4 group as the training set, and the dataset as the testing set. For LODO analysis, the AUC value for a given dataset is obtained using the combined dataset in V1-V2 or V4 group except the dataset as the training set and the study as the testing set. Rank row is calculated using the following steps (1) for each dataset, rank each method among all methods based on AUC values (larger AUC has a smaller rank); (2) calculate the average of the ranks across datasets for each method.


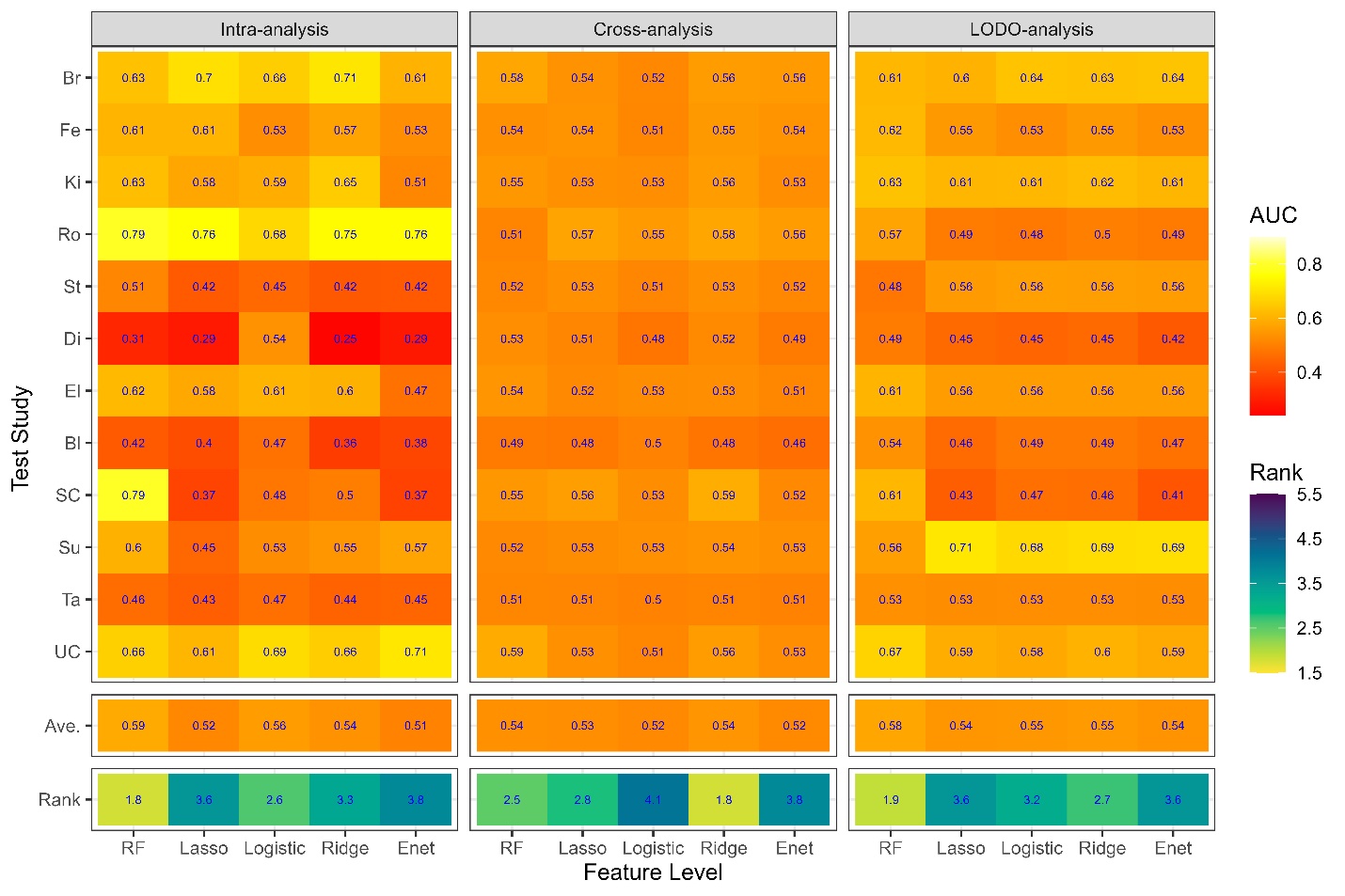


**Figure S6.** Assessment prediction performance of classifiers using the genus features with CLR transformation. Rank row is calculated using the following steps (1) for each dataset, rank each method among all methods based on AUC values (larger AUC has a smaller rank); (2) calculate the average of the ranks across datasets for each method.


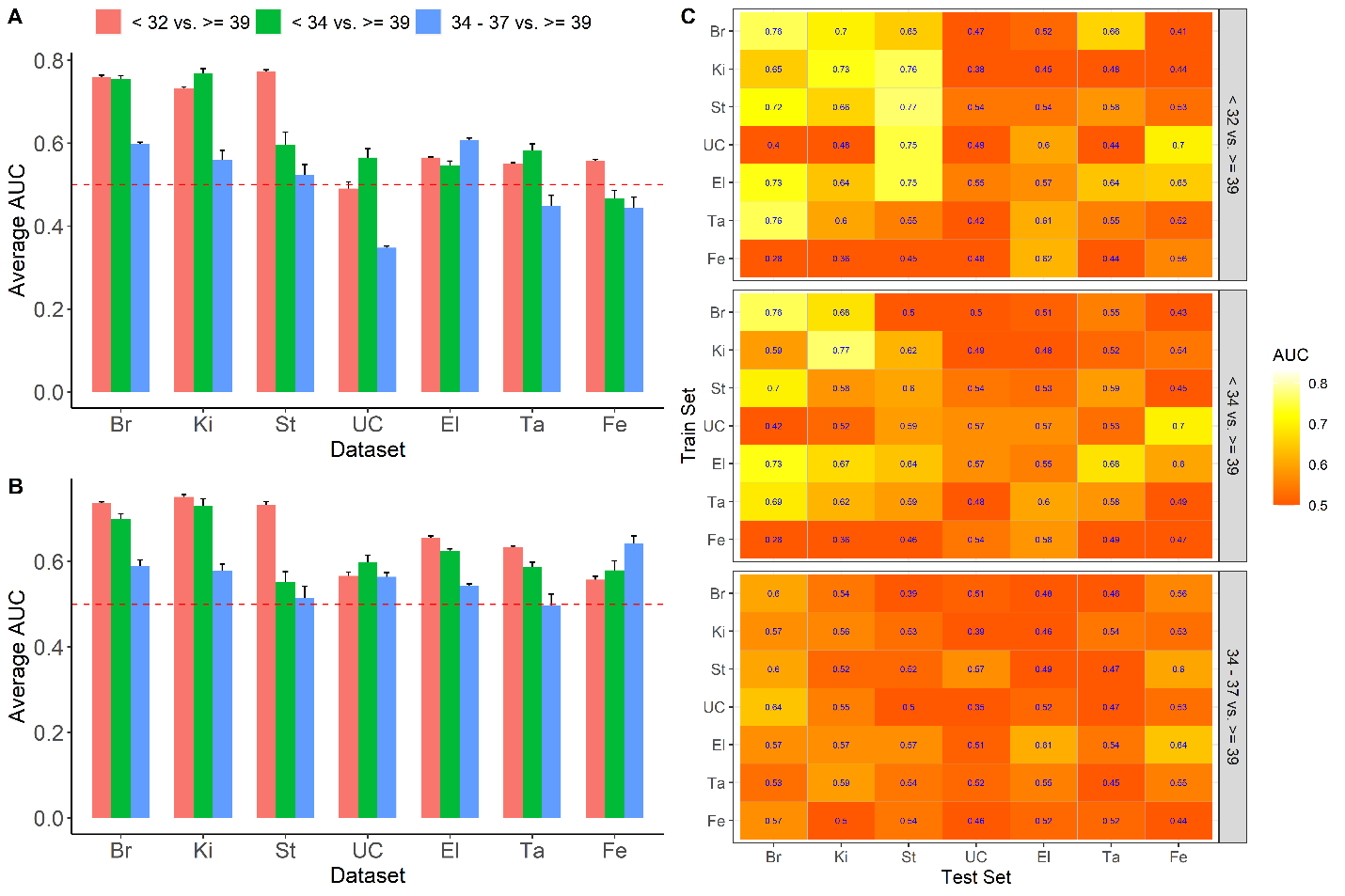


**Figure S7.** Assessment of prediction performance for different preterm birth groups using intra-dataset analysis (A), combined-dataset analysis (B) and cross-dataset analysis (C) using proportional abundance data.


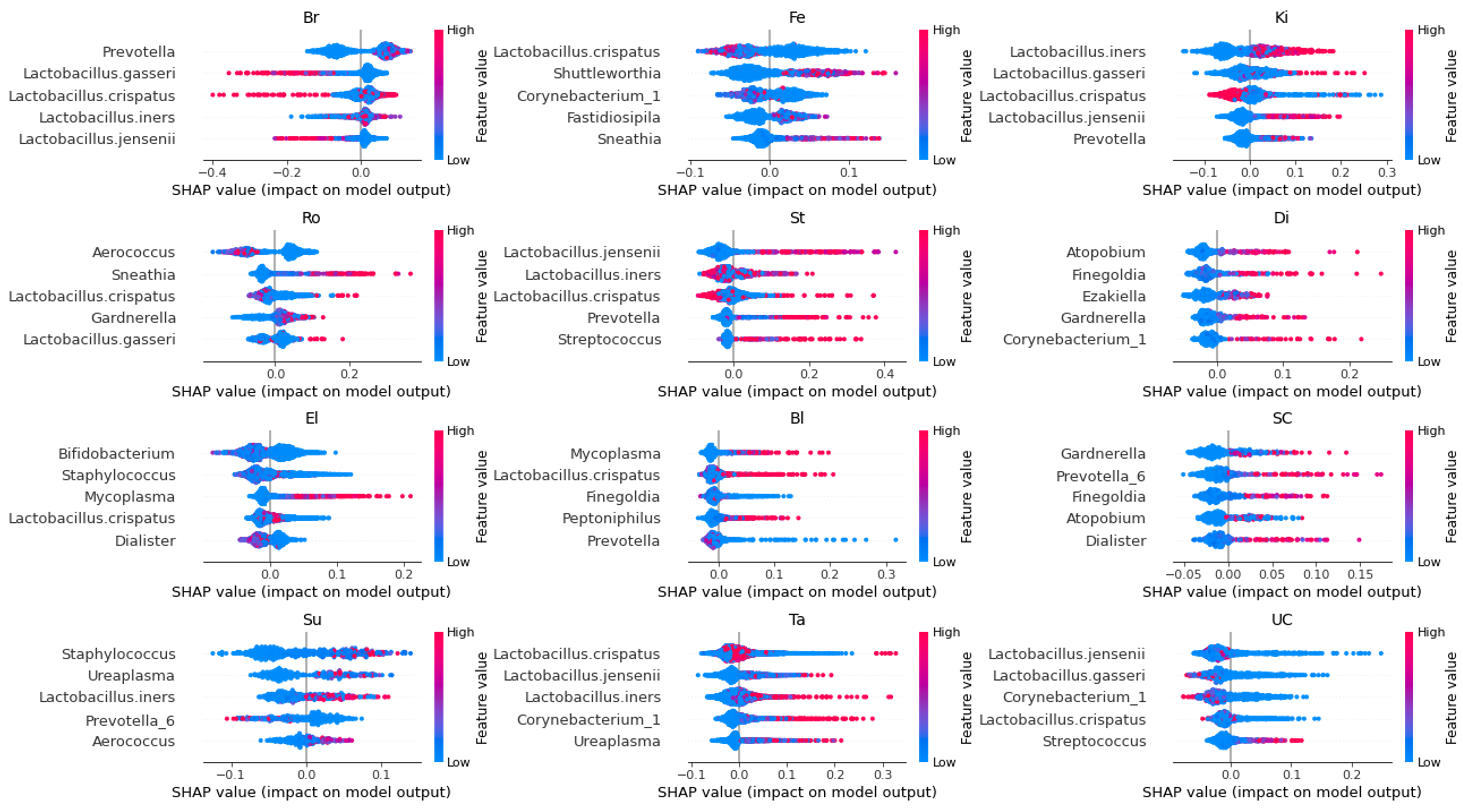


**Figure S8.** SHAP summary plots for each study for random forests trained on genus-level proportional data. The five most important features for each study are shown. The data points are colored by the relative abundance of the taxa. Positive SHAP values indicate a higher likelihood of preterm birth and negative SHAP values indicate a lower likelihood of preterm birth in comparison to the average prediction.


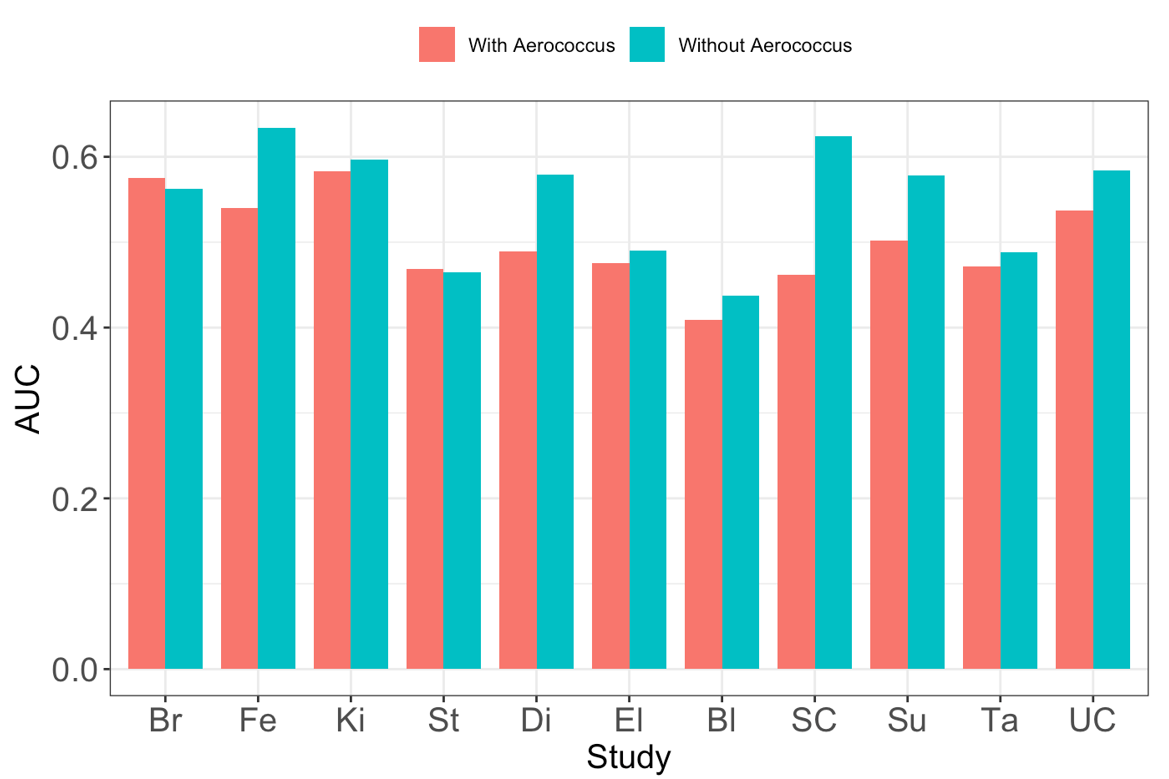


**Figure S9.** Average AUC with and without Aerococcus included as a feature for ten random forest models trained on genus-level CLR data from the Ro study and tested on all other studies.


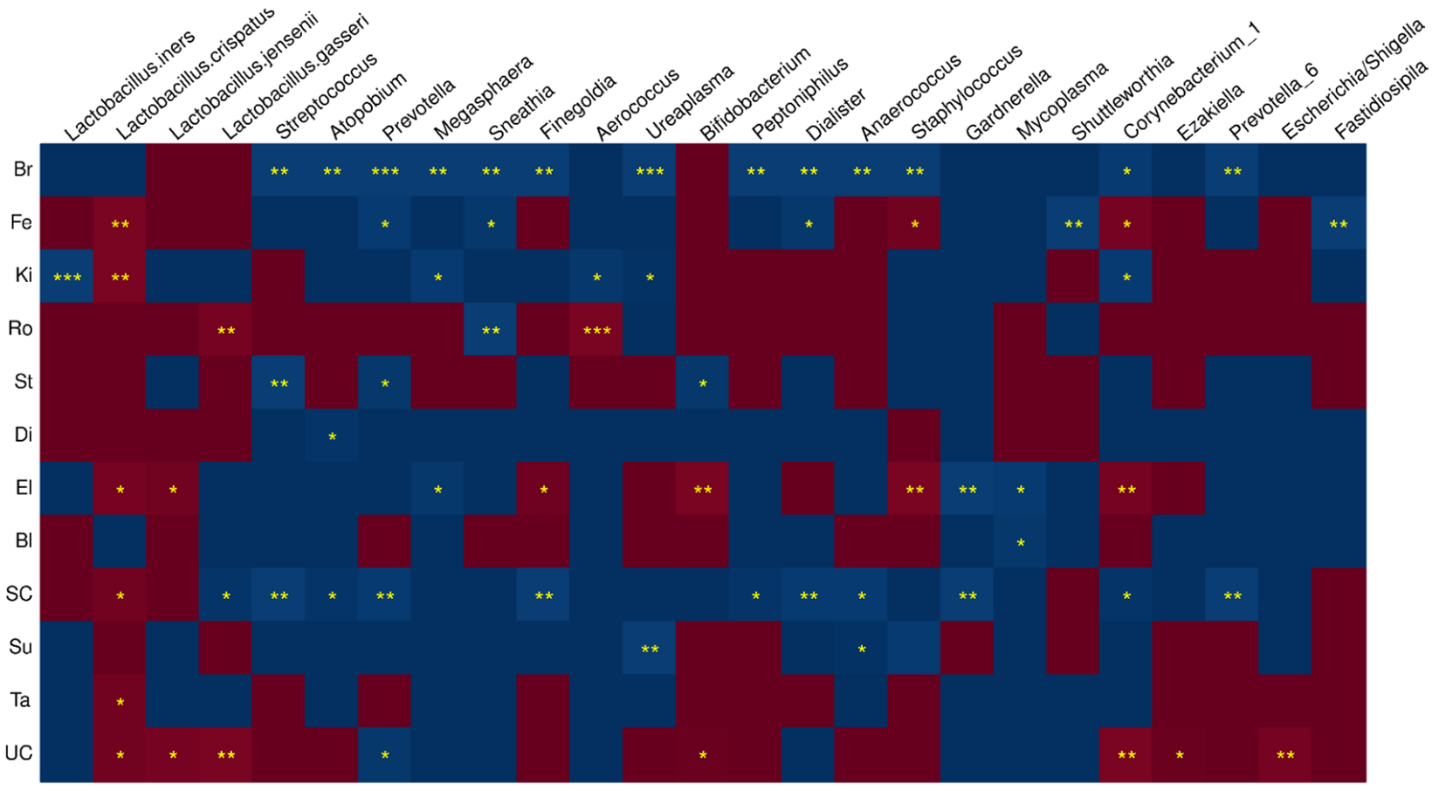


**Figure S10.** Dataset-specific differential abundance analysis for each genus/species using a one-sided Wilcoxon rank-sum test. A cell is in red color if the average relative abundance of a genus/species in term birth is larger than the average relative abundance in preterm birth, otherwise in blue color. The unadjusted p-value significant code: ‘***’: 0.001, ‘**’: 0.01, ‘*’: 0.05.


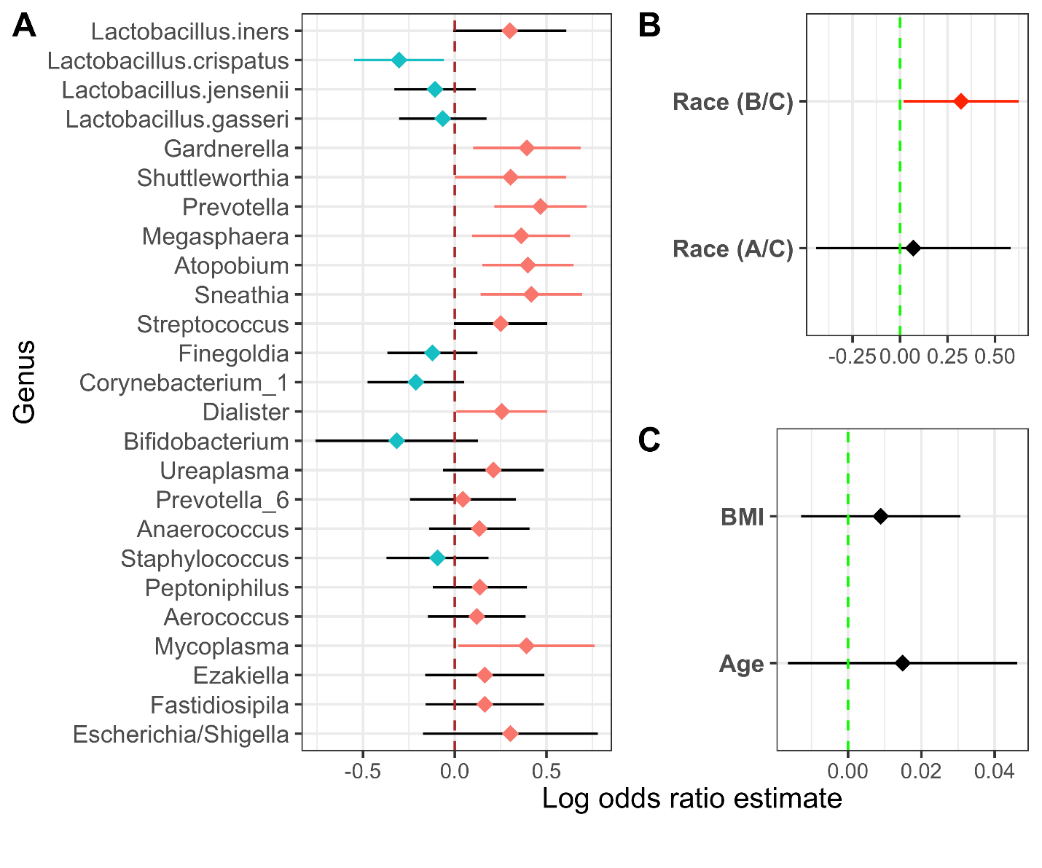


**Figure S11.** Cross-dataset differential abundance analysis using a generalized linear mixed model with covariates. The genus and race effects were estimated using 11 datasets with a model including race covariate. The BMI and age effects were estimated using 8 datasets with a model including race, BMI and age. Point estimates less than 0 are shown as blue points and greater than 0 are shown as red points. Confident intervals less than 0 are shown as blue bars and greater than 0 are shown as red bars.


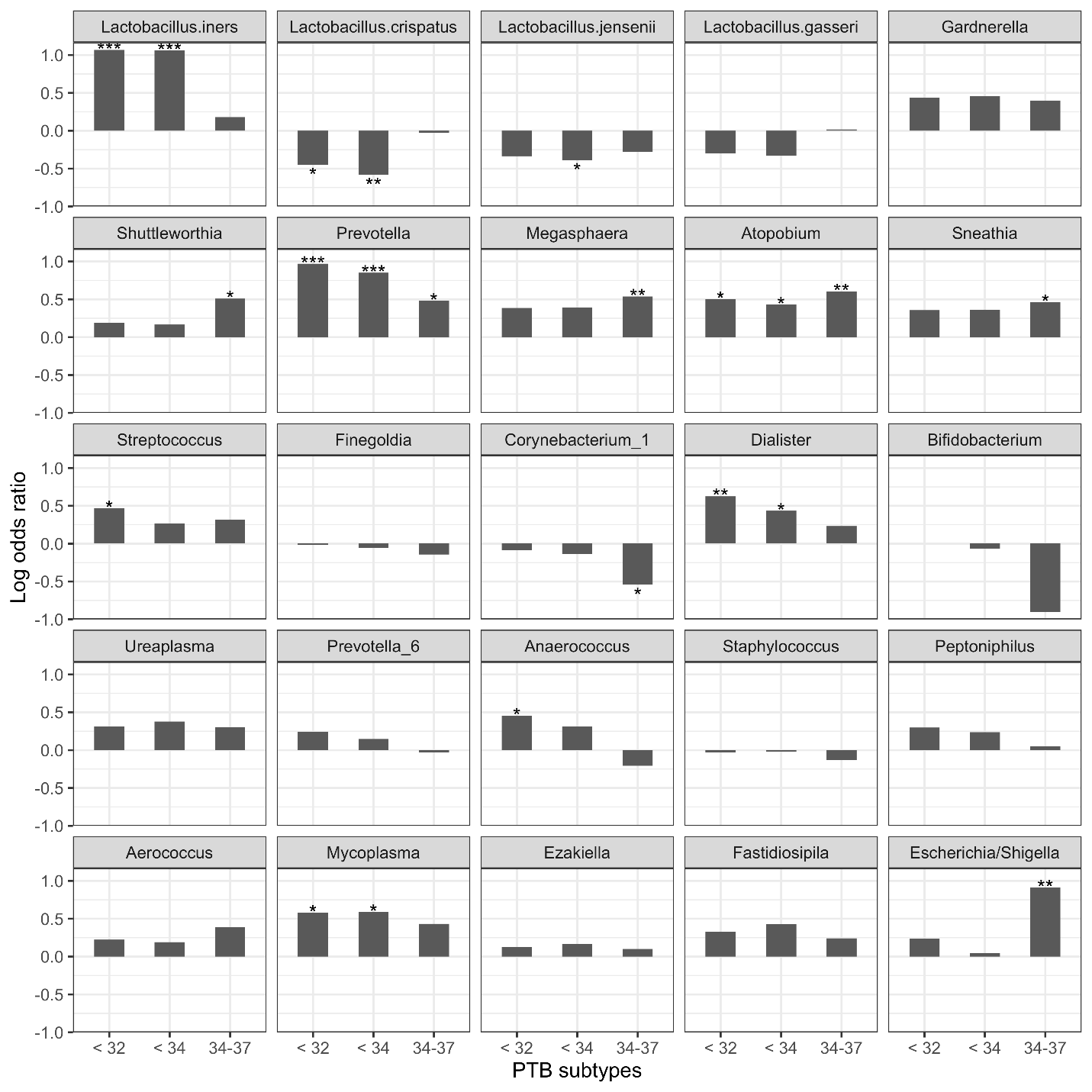


**Figure S12.** Average estimates of log odd ratio for each PTB subgroup versus late term birth using the generalized linear mixed model. The asterisk shows the significant level of the combined p-values: *: p-value < 0.05; **: p-value < 0.01; ***: p-value < 0.001.


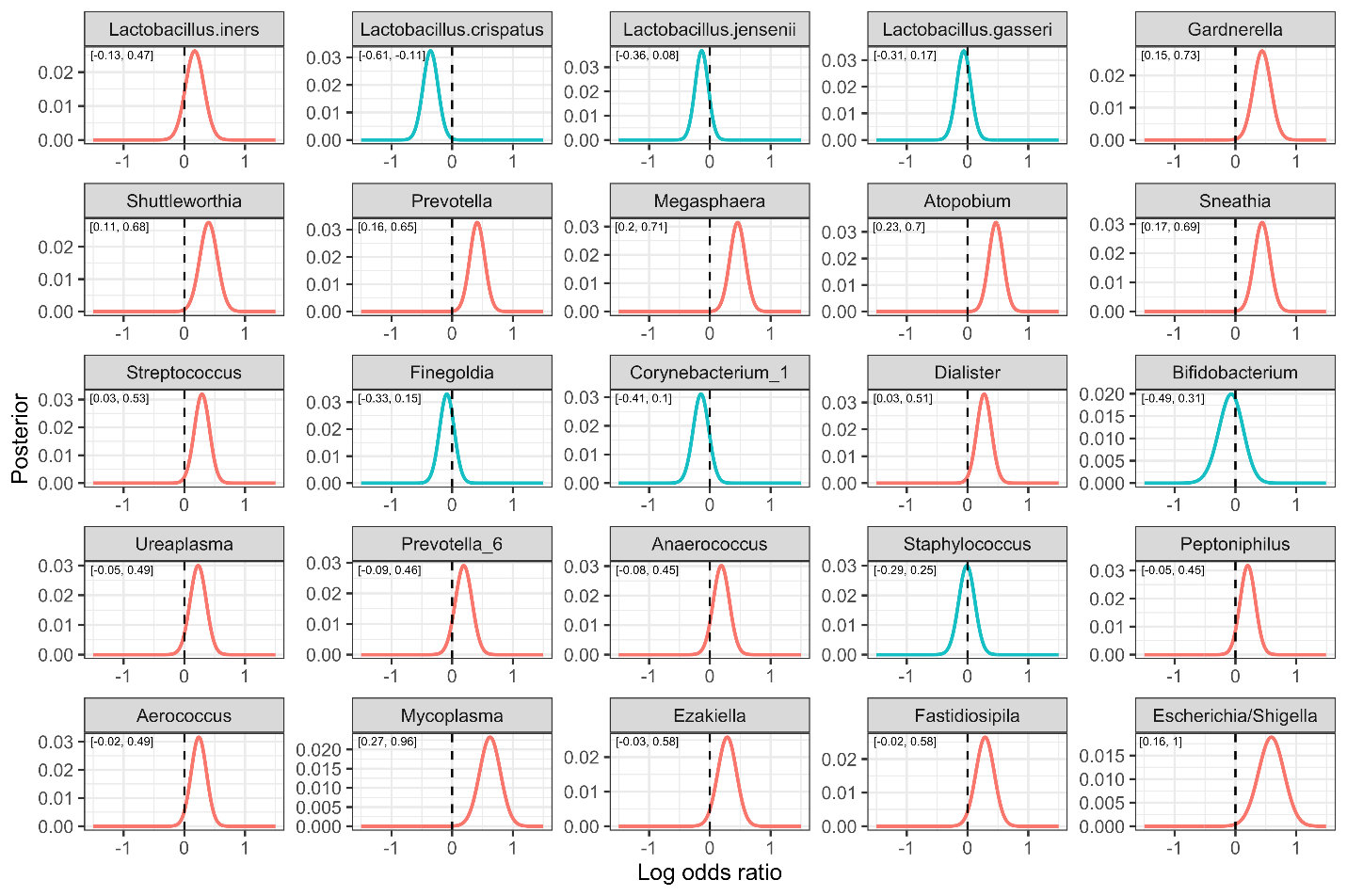


**Figure S13.** Posterior distribution of log odds ratio using a Bayesian approach stepwise method. Maximum a posteriori (MAP) estimates less than 0 are shown as blue lines and larger than 0 are shown as red lines. The 95% credible intervals are shown in the left top corner.
