# Supplementary material for: Meta-analysis reveals the vaginal microbiome is a better predictor of earlier than later preterm birth
Source: BMC Biol. 2023 Sep 25;21:199. doi: 10.1186/s12915-023-01702-2 (PMC10518966; doi:10.1186/s12915-023-01702-2)
Supplement: Supplementary file 4 — Additional file 4: Supplementary Methods. Details about classifiers used in the meta-analysis and the Bayesian approach. [file 12915_2023_1702_MOESM4_ESM.pdf]

Supplementary Methods

Classifiers

We implemented the following classifiers in the machine learning framework and compared their performance: (1) random forest; (2) logistic regression; (3) L1-regularized (LASSO) logistic regression; (4) L2-regularized (Ridge) logistic regression; (5) Elastic net regularized logistic regression. Random forest was implemented using the randomForest R package with 1000 trees. The hyperparameter 'mtry' (number of variables randomly sampled as candidates at each split) was tuned using the caret R package with repeated cross-validation. Logistic regression was implemented using the glm function in R. The regularized logistic regression models were implemented using the glmnet R package. The hyperparameter 'lambda' in the LASSO and Ridge models was tuned using the cv.glment function. The hyperparameters 'alpha' and 'lambda' were tuned using the caret R package with repeated cross-validation.

Bayesian Approach

Let $G = 1$ if a genus is present (relative abundance > 0.001) and 0 if absent, $B = 1$ if a women has preterm birth (PTB) and 0 if term birth. We can have the conditional probability of PTB giving a genus is absent for dataset i to be

$$p_i(B=1|G=0) = p_i^0 = \frac{u_i}{1+u_i}$$

where u_i is the odds of PTB giving a genus is absent for dataset i.
Define r as the odds ratio between a genus is present and absent and it is the same for different datasets. Thus, the conditional probability of PTB giving a genus is present for dataset i can be written as

$$p_i(B=1|G=1) = p_i^1 = \frac{u_i r}{1+u_i r}$$

We assume both u_i and r have prior distributions, $p(u_i)$ and $p(r)$, respectively. We are interested in calculating the posterior distribution of r. Given dataset i, let N_i is the total number of subjects in study i, n_i is the number of subjects that with certain genus present. M_i is the number of PTB subjects in study i, m_i is the number of PTB subjects that with certain genus present. Thus, we can have the likelihood function

$$\begin{aligned}
&p(N_i, n_i, M_i, m_i | u_i, r) \\
&= (p_i^0)^{M_i - m_i}(1-p_i^0)^{(N_i - n_i)-(M_i - m_i)}(p_i^1)^{m_i}(1-p_i^1)^{n_i - m_i} \\
&= \frac{u_i^{M_i - m_i}}{(1+u_i)^{N_i - n_i}} * \frac{(u_i r)^{m_i}}{(1+u_i r)^{n_i}} \\
&= \frac{u_i^{M_i} r^{m_i}}{(1+u_i)^{N_i - n_i}(1+u_i r)^{n_i}}
\end{aligned} \quad \textbf{(1)}$$

Thus, the posterior distribution of u_i and r can be written as

$$\begin{aligned}
p(u_i, r | N_i, n_i, M_i, m_i) &= \frac{p(N_i, n_i, M_i, m_i | u_i, r) p(u_i, r)}{p(N_i, n_i, M_i, m_i)} \\
&= \frac{p(N_i, n_i, M_i, m_i | u_i, r) p(u_i) p(r)}{\int_{u_i}\int_r p(N_i, n_i, M_i, m_i | u_i, r) p(u_i) p(r)}
\end{aligned} \quad \textbf{(2)}$$

Furthermore, we can integrate out u_i and obtain the posterior distribution for odds ratio r,

$$\begin{aligned}
p(r | N_i, n_i, M_i, m_i) &= \int_{u_i} p(u_i, r | N_i, n_i, M_i, m_i) \\
&= \frac{p(r)\int_{u_i} p(N_i, n_i, M_i, m_i | u_i, r) p(u_i)}{\int_{u_i}\int_r p(N_i, n_i, M_i, m_i | u_i, r) p(u_i) p(r)}
\end{aligned} \quad \textbf{(3)}$$

We assume the $\log(u_i)$ follows a uniform prior distribution for each dataset. For r, we let the first dataset has the uniform prior distribution, then calculate the posterior distribution of r. Next we let the posterior distribution of the odds ratio from the first dataset be the prior distribution for the second dataset, and update the posterior distribution of r. Repeated the process until the last dataset and obtain the final posterior distribution of r.
